# Pharmacogenomics of poor drug metabolism in Greyhounds: Cytochrome P450 (CYP) 2B11 genetic variation, breed distribution, and functional characterization

**DOI:** 10.1038/s41598-019-56660-z

**Published:** 2020-01-09

**Authors:** Stephanie E. Martinez, Marie C. Andresen, Zhaohui Zhu, Ioannis Papageorgiou, Michael H. Court

**Affiliations:** 1 Comparative Pharmacogenomics Laboratory, Program in Individualized Medicine (PrIMe), Department of Veterinary Clinical Sciences, College of Veterinary Medicine, Washington State University, Pullman, Washington United States of America; 2 Present Address: Department of Pathology, Immunology and Laboratory Medicine, College of Medicine, Diabetes Institute, University of Florida, Gainesville, Florida United States of America

**Keywords:** Pharmacogenetics, Pharmacogenomics, Mutation, Genetics research

## Abstract

Greyhounds recover more slowly from certain injectable anesthetics than other dog breeds. Previous studies implicate cytochrome P450 (CYP) 2B11 as an important clearance mechanism for these drugs and suggest Greyhounds are deficient in CYP2B11. However, no *CYP2B11* gene mutations have been identified that explain this deficiency in Greyhounds. The objectives of this study were to provide additional evidence for CYP2B11 deficiency in Greyhounds, determine the mechanisms underlying this deficiency, and identify *CYP2B11* mutations that contribute to this phenotype in Greyhounds. Greyhound livers metabolized CYP2B11 substrates slower, possessed lower CYP2B11 protein abundance, but had similar or higher mRNA expression than other breeds. Gene resequencing identified three *CYP2B11* haplotypes, H1 (reference), H2, and H3 that were differentiated by mutations in the gene 3′-untranslated region (3′-UTR). Compared with 63 other dog breeds, Greyhounds had the highest *CYP2B11*-H3 allele frequency, while *CYP2B11*-H2 was widely distributed across most breeds. Using 3′-UTR luciferase reporter constructs, *CYP2B11*-H3 showed markedly lower gene expression (over 70%) compared to *CYP2B11*-H1 while *CYP2B11*-H2 expression was intermediate. Truncated mRNA transcripts were observed in *CYP2B11*-H2 and *CYP2B11*-H3 but not *CYP2B11*-H1 transfected cells. Our results implicate *CYP2B11* 3′-UTR mutations as a cause of decreased CYP2B11 enzyme expression in Greyhounds through reduced translational efficiency.

## Introduction

Although the genetic causes underlying racial, ethnic, and population differences in drug disposition and response have been extensively studied in people^1^, relatively little is currently known regarding the source of variable drug effects among different breeds of domestic dog. The only example so far in which the mechanism of a dog breed drug sensitivity has been determined are the Collies and related herding breeds, which were shown to be sensitive to p-glycoprotein substrates because of a 4-base pair deletion mutation in the gene encoding this transporter^2^. Another group of dog breeds that have been reported to display significantly different drug response compared with other breed groups are the “Sighthounds”. Sighthounds (also known as “Gazehounds”) are so-called because they were bred to hunt prey primarily by sight (or gaze), rather than by scent, as is typical of the “Scent hound” grouping of breeds. Modern Greyhounds are a prototypical example of a Sighthound dog breed that have been bred for over 150 years for hunting, coursing, track racing and other purposes^3^. It is well known among veterinarians, owners and breeders of Greyhounds (and related Sighthound breeds) that many of these dogs are likely to recover more slowly after receiving certain injectable anesthetic drugs compared with other dog breeds^4–6^. These drugs include several thiobarbiturates (thiopental and thiamylal), as well as propofol, which has largely replaced the thiobarbiturates for routine induction of anesthesia in dogs and humans.

Initially, this anesthetic sensitivity was thought to be a consequence of the naturally low body fat content of the Greyhound breed, which could limit redistribution of lipophilic anesthetic drugs from the brain into peripheral fatty tissues and delay the return of consciousness^4–6^. However, a series of elegant studies subsequently implicated poor drug metabolism as a major culprit. Pharmacokinetic studies demonstrated slower elimination of thiopental, thiamylal and propofol in Greyhounds compared with mixed-breed dogs^7,8^. Furthermore, both pentobarbital and methohexital, oxybarbiturate anesthetics with similar lipophilicity to thiobarbiturates but slightly different chemical structures, displayed similar recovery times and plasma pharmacokinetic parameters in Greyhounds compared with mixed-breed dogs^7^. Finally, treatment of Greyhounds with the cytochrome P450 (CYP) enzyme inducer, phenobarbital, enhanced thiopental clearance and reduced recovery times^9^, while treatment with the CYP inhibitor, chloramphenicol, reduced propofol clearance and prolonged recovery times^10^. These studies implicate a major role for CYP in the elimination of these drugs in dogs and suggest that Greyhounds could be deficient in one (or more) CYP enzymes.

Propofol 4-hydroxylation is the major rate limiting step in the clearance of propofol in dogs^11^. A previous study in our laboratory demonstrated reduced propofol 4-hydroxylation by liver microsomes obtained from Greyhounds, compared to Beagle (a breed commonly used in pharmaceutical research and development) and mixed-breed dog liver microsomes^12^. An additional study using CYP isoform-selective chemical and antibody inhibitors implicated an important role for CYP2B11 (the canine ortholog of human CYP2B6) in propofol hydroxylation by canine liver microsomes and suggested this isoform may be deficient in Greyhounds^13^. However, as of yet, there is no direct evidence that CYP2B11 selectively metabolizes propofol, such as through reaction phenotyping using recombinant canine CYPs. Furthermore, no mutations in the gene encoding the CYP2B11 protein, *CYP2B11* (also called *CYP2B6*, NCBI gene ID 474177), have been identified in Greyhounds that could explain this deficiency.

The primary objectives of this study were to provide further evidence that CYP2B11 is deficient in Greyhounds, determine the genetic mechanisms underlying this deficiency, and identify *CYP2B11* gene mutations that may contribute to poor drug metabolism in Greyhounds. We also explored the distribution of the identified *CYP2B11* gene mutations across dog breeds, hypothesizing that they would be more prevalent in Greyhounds and closely related breeds within the Sighthound group of dog breeds compared to non-Sighthound breeds.

## Results

### Dog breed differences in hepatic CYP probe activities

**Figure 1:**
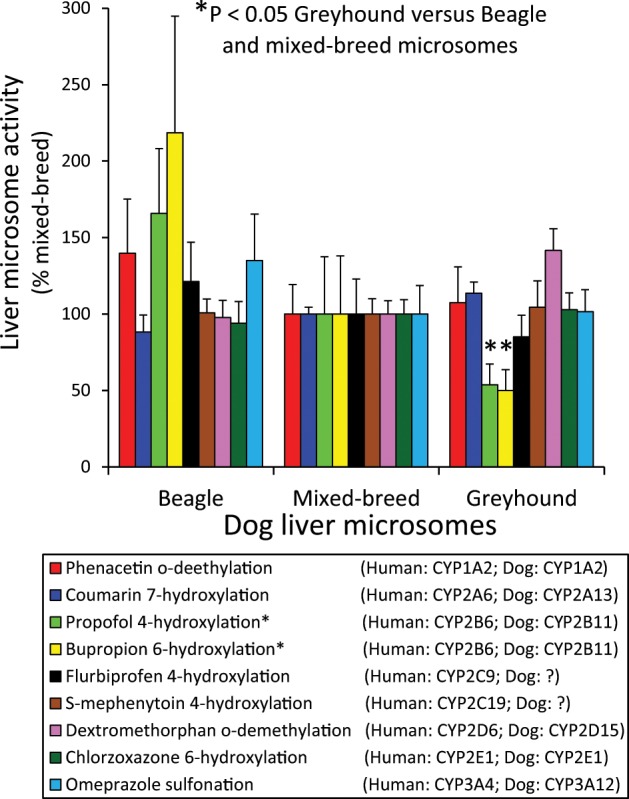
Breed differences in liver microsome CYP marker activities. CYP activities selective for the major drug metabolizing CYP enzymes were measured using liver microsomes obtained from Beagles (n = 5), mixed-breed dogs (n = 5) and Greyhounds (n = 5). Bars represent the mean and standard error of the activity values for individual Beagle and Greyhound liver microsomes expressed as a percentage of the mean activity of mixed-breed dog liver microsomes. *P < 0.05 by Student’s *t*-test on log transformed data comparing Greyhound dog liver activities with Beagle and mixed-breed dog liver activities. Samples from Greyhound dogs were identified by their owners as dogs registered with the National Greyhound Association bred for racing.

Eight enzyme activities commonly used as isoform-selective probes for the major drug metabolizing CYPs in humans were measured in Greyhound, Beagle and mixed-breed dog liver microsomes (n = 5 livers per breed) to explore possible breed-related differences in hepatic CYP metabolism. Results were compared to an activity (propofol 4-hydroxylation) previously demonstrated to be lower in Greyhound livers compared with livers from other dog breeds^13^. As shown in Fig. 1, average propofol 4-hydroxylation, and bupropion 6-hydroxylation were lower in Greyhound liver microsomes (P < 0.05, Student’s *t*-test) relative to mixed-breed and Beagle liver microsomes. On the other hand, average activities for all other CYP probes measured in Greyhound microsomes were similar to, or in the case of dextromethorphan *O*-demethylation activities somewhat higher than, activities for mixed-breed and Beagle microsomes.

### Propofol, bupropion, and omeprazole reaction phenotyping

**Figure 2:**
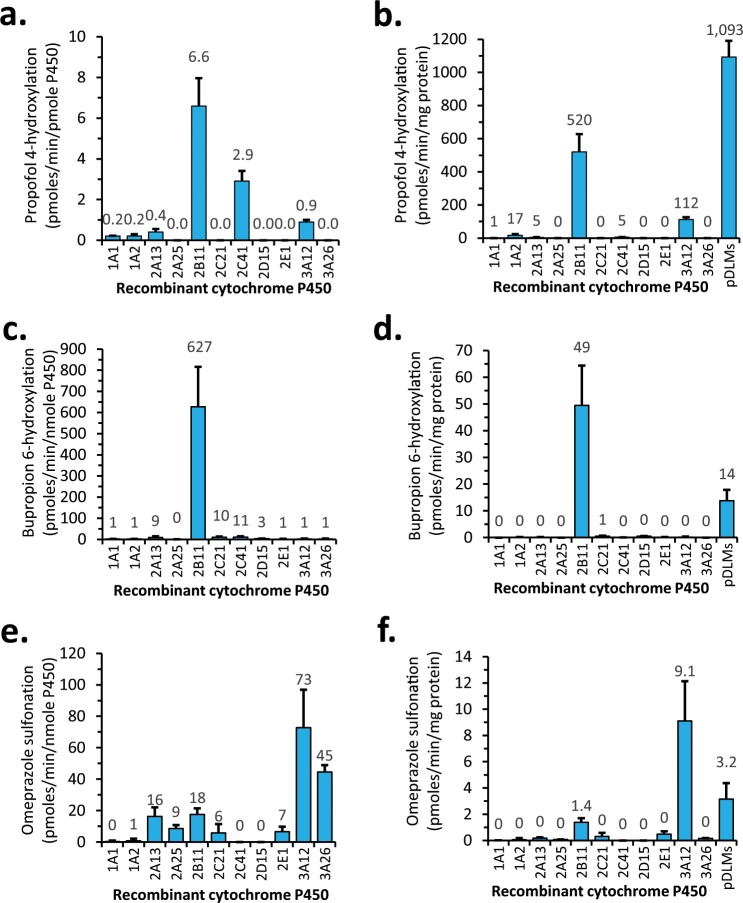
Reaction phenotyping using a panel of recombinant canine CYP enzymes. The rates of propofol 4-hydroxylation (**a,b**) bupropion 6-hydroxylation (**c,d**) and omeprazole sulfonation (**e,f**) were determined using a panel of 11 recombinant canine CYP enzymes. Results are shown after normalization to incubation time and recombinant CYP concentration in each reaction (**a,c,e**) as well as after extrapolation of activities to microsomes using the reported average molar concentration of each CYP in canine liver microsomes (**b,d,f**). Details are provided in Materials and Methods section. Activities for pooled dog liver microsomes (pDLMs) normalized to microsomal protein content are also shown for comparison. Bars represent the mean and standard deviation of 3 independent replicate experiments.

Reaction phenotyping with recombinant canine CYP enzymes was then used to confirm the identity of the canine CYPs responsible for the two activities decreased in Greyhound microsomes (propofol 4-hydroxylation and bupropion 6-hydroxylation). We also verified the specificity of omeprazole sulfonation as a canine CYP3A12 probe, since another commonly used human CYP3A probe activity (midazolam 1′-hydroxylation) was reported to be primarily mediated by canine CYP2B11^14^. All 8 commercially available recombinant canine hepatic CYPs were evaluated as well as an additional 3 recombinant drug metabolizing CYPs (CYP2A13, CYP2A25, and CYP2E1) that were expressed in our laboratory. Measured specific activities for each recombinant CYP were also normalized using the average canine liver microsome abundance of each CYP to enable direct comparison to activities measured using pooled dog liver microsomes. As shown in Fig. 2a, CYP2B11 displayed the greatest propofol 4-hydroxylation activity; CYP2C41 and CYP3A12 had moderate activities (44% and 14% of CYP2B11, respectively), while all other CYPs showed minimal activity. After extrapolation of CYP activities using canine hepatic abundance estimates (Fig. 2b), CYP2B11 remained the most active enzyme, which was approximately 50% of the propofol 4-hydroxylation activity of pooled dog liver microsomes. Some abundance-corrected activity was also observed for CYP3A12 (22% of CYP2B11), while activities for other CYPs were negligible. Bupropion 6-hydroxylation was mediated exclusively by CYP2B11 (Fig. 2c) with negligible activities observed for all other CYPs tested. After extrapolation using average liver abundance estimates, CYP2B11 bupropion 6-hydroxylation activity was more than 3 times that of pooled dog liver microsomes, while all other CYPs showed negligible activity relative to pooled dog liver microsomes (Fig. 2d). Finally, substantial omeprazole sulfonation activity was observed for both canine CYP3A isoforms (CYP3A12 and CYP3A26). CYP3A12 was the most active, about 50% higher than CYP3A26, and more than 4 times higher than other isoforms (Fig. 2e). After hepatic abundance correction, CYP3A12 was the predominant enzyme, with almost three times the omeprazole sulfonation activity of pooled liver microsomes (Fig. 2f). Some abundance corrected activity was also observed for CYP2B11 (15% of CYP3A12), but not for other CYPs.

These results were further confirmed by evaluating the strength of correlation between CYP probe activities and CYP1A, CYP2B11 and CYP3A protein content measured by semi-quantitative immunoblotting in the same set of dog liver microsomes. Spearman correlation coefficients and their respective P-values are shown in Table 1. CYP2B11 protein content correlated strongly with both bupropion 6-hydroxylation (Rs = 0.73, P = 0.002) and propofol 4-hydroxylation (Rs = 0.70, P = 0.003), but not with any other activity (P > 0.05). Similarly, CYP3A protein content correlated only with omeprazole sulfonation (Rs = 0.86, P < 0.0001) and CYP1A protein content correlated only with phenacetin-O-deethylation (Rs = 0.59, P = 0.02).

**Table 1:** Correlation of CYP isoform protein content with CYP marker activities measured in dog liver microsomes.

Enzyme marker activity	Attributed CYPs		Spearman correlation coefficient (P-value)		
	Human	Dog	CYP1A protein	CYP2B11 protein	CYP3A protein
Phenacetin-*o*-deethylation	CYP1A2	CYP1A2	0.59 (0.02*)	0.30 (0.28)	0.10 (0.71)
Coumarin 7-hydroxylation	CYP2A6	CYP2A13	−0.29 (0.29)	−0.35 (0.20)	0.06 (0.83)
Propofol 4-hydroxylation	CYP2B6	CYP2B11	0.32 (0.24)	0.70 (0.003*)	0.18 (0.51)
Bupropion 6-hydroxylation	CYP2B6	CYP2B11	0.25 (0.36)	0.73 (0.002*)	0.00 (1.00)
Flurbiprofen hydroxylation	CYP2C9	?	0.41 (0.13)	0.42 (0.12)	0.14 (0.61)
S-mephenytoin 4-hydroxylation	CYP2C19	?	0.38 (0.16)	0.28 (0.31)	0.15 (0.56)
Dextromethorphan *o*-demethylation	CYP2D6	CYP2D15	−0.31 (0.26)	−0.47 (0.07)	−0.05 (0.84)
Chlorzoxazone 6-hydroxylation	CYP2E1	CYP2E1	0.06 (0.81)	−0.10 (0.71)	−0.04 (0.87)
Omeprazole sulfonation	CYP3A4	CYP3A12	0.13 (0.64)	0.35 (0.20)	0.86 (<0.001*)

CYP isoform protein content (determined by immunoblotting) and CYP marker activities were measured in the same set of dog liver microsomes (n = 15) and correlated. Shown are the Spearman correlation coefficients and associated P-values (*P < 0.05). Also shown are the human and canine CYP isoforms that have been attributed to each activity by reaction phenotyping. (?) – No evidence yet to identify the dog CYP isoform responsible for this activity.

### Dog breed differences in hepatic CYP2B11 protein and mRNA

**Figure 3:**
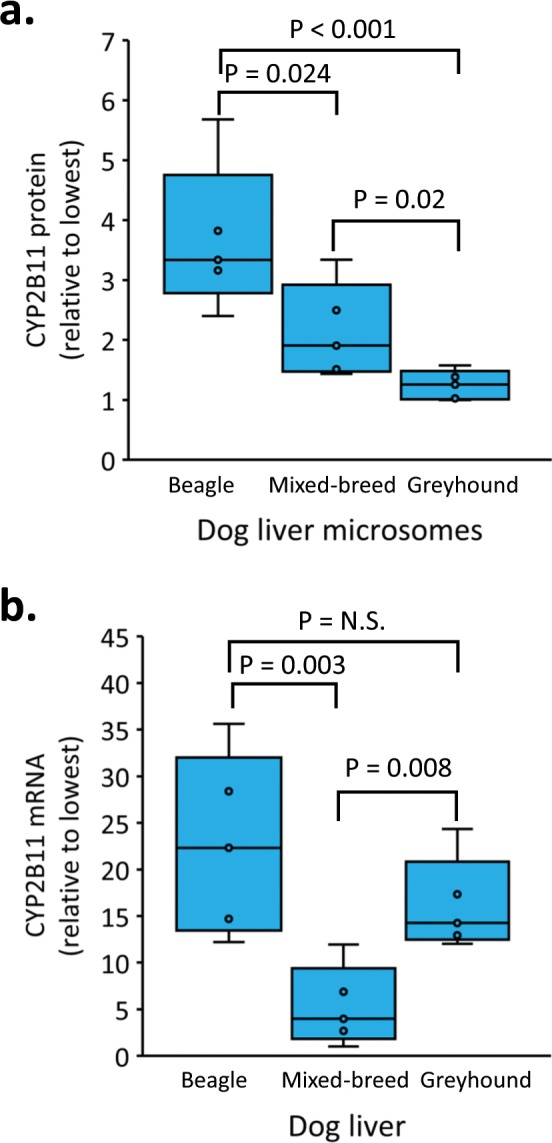
Breed differences in CYP2B11 protein and mRNA. Microsomal CYP2B11 protein content (**a**) and *CYP2B11* mRNA abundance (**b**) were measured in the same set of livers obtained from Beagles (n = 5), mixed-breed dogs (n = 5) and Greyhounds (n = 5). Data are expressed relative to the liver with the lowest value. Shown are box and whiskers plots summarizing data for individual dogs in each breed group. Significant differences between breed groups were identified by ANOVA on log transformed data (P < 0.05) for both CYP2B11 protein and mRNA. Shown for each set of data are the P-values for *post hoc* pairwise multiple comparisons testing (Holm-Sidak method).

Microsomal CYP2B11 protein content and *CYP2B11* mRNA abundance were measured in the same set of Greyhound, Beagle and mixed-breed dog liver samples (n = 5 livers per breed). As shown in Fig. 3a, significant breed associated differences in CYP2B11 content were observed (P < 0.001, ANOVA). Greyhound livers showed the lowest content, Beagle livers had the highest content, and mixed-breed livers were intermediate. On the other hand, *CYP2B11* mRNA abundance in Greyhound livers was similar to Beagle livers (P > 0.05, Holm-Sidak test) and substantially higher than mixed-breed livers (P = 0.008; Holm-Sidak test) (Fig. 3b).

### Identification of *CYP2B11* genetic polymorphisms

Selected regions of the *CYP2B11* gene, including the 5′-enhancer (to ~2,000 bp upstream), all 9 exons, and the complete 3′-untranslated region (UTR) were sequenced using DNA obtained from 13 Greyhounds, including the 5 Greyhounds used for liver samples. Sequence variants were identified by comparison to the current canine reference sequence (CanFam3.1) and compared to polymorphisms identified by analysis of publicly available whole genome sequence data from another 45 dogs representing 45 different breeds. Identified polymorphisms and the genotypes of individual dogs are given in Supplementary Table S1. These data are summarized as variant allele frequencies (with 95% confidence intervals) for the 13 Greyhounds and the 45 dogs from other breeds in Table 2. Nine genetic polymorphisms were identified, three of which were found in the dbSNP public database (rs21894687, rs852076551, and rs850924485). One polymorphism was located in the 5′-enhancer region (c.-489 G/A), one polymorphism was a synonymous SNP in exon 7 (c.966G/A), while the remaining 7 polymorphisms were clustered together in the 3′-UTR from cDNA positions 1913 to 2536. Allele frequencies for all but one of the 3′-UTR polymorphisms were more than 2-fold higher in the 13 Greyhounds compared to the 45 other dogs. One 3′-UTR polymorphism (c.2498G/T) was not found in any of the 13 Greyhounds evaluated.

**Table 2:** *CYP2B11* genetic polymorphisms and allele frequencies.

	Genetic polymorphism								
	#1	#2	#3	#4	#5	#6	#7	#8	#9
Position^a^	112817078	112828499	112832580	112832619	112832805	112832834	112832951	112833166	112833204
Reference allele	G	G	TCA	C	TG	G	A	G	G
Alternate allele	A	A	TCCA	T	CA	A	G	T	C
dbSNP (v.146) ID	—	rs21894687	rs852076551	—	—	—	—	rs850924485	—
Location	5′-enhancer	Exon 7	3′UTR	3′UTR	3′UTR	3′UTR	3′UTR	3′UTR	3′UTR
Protein	—	p.Glu322Glu	—	—	—	—	—	—	—
cDNA	c.-489_G/A	c.966_G/A	c.1913_TCA/TCCA	c.1952_C/T	c.2137_TG/CA	c.2166_G/A	c.2283_A/G	c.2498_G/T	c.2536_G/C
	**Allele frequencies (95% C.I.)**								
Greyhounds (N = 13)	0.50	0.50	0.50	0.19	0.50	0.50	0.50	1.0	0.50
	(0.30–0.70)	(0.30–0.70)	(0.30–0.70)	(0.09–0.38)	(0.30–0.70)	(0.30–0.70)	(0.30–0.70)	(0.87–1.0)	(0.30–0.70)
Other breeds (N = 45)	0.24	0.34	0.20	0.03	0.18	0.18	0.18	0.86	0.18
	(0.17–0.34)	(0.25–0.45)	(0.13–0.29)	(0.01–0.09)	(0.11–0.27)	(0.11–0.27)	(0.11–0.27)	(0.77–0.91)	(0.09–0.23)

**Figure 4:**
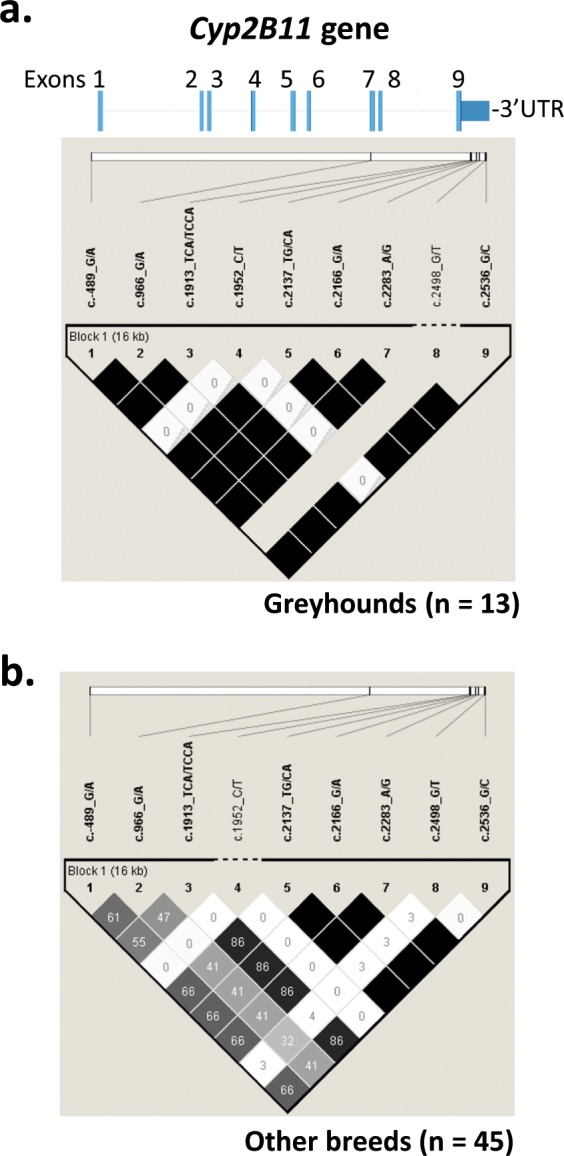
Linkage disequilibrium across the *CYP2B11* gene. Results of genotype association analysis conducted using the Haploview program^40^. Shown are the locations of polymorphic sites identified in the 5′-enhancer, exons 1 to 9, and 3′UTR in 13 Greyhounds (**a**) and single dogs sampled from 45 different breeds (**b**). Below each set of polymorphisms are matrices of linkage disequilibrium *r*^2^ values (as a percent) for pairwise comparisons. All black squares indicate complete linkage (*r*^2^ = 100%). c.1952 C/T (#4) was not associated (*r*^2^ = 0%) with any other polymorphism genotyped in both Greyhounds and non-Greyhound dogs. c.2498 G/T (#8) was invariant in all Greyhounds genotyped. The genetic polymorphism labels used here (#1 to #9) correspond to the labels used in Tables 2 and 3. Samples from Greyhound dogs were identified by their owners as dogs registered with the National Greyhound Association bred for racing.

Genetic polymorphisms located in the *CYP2B11* 5′-enhancer (to ~2,000 bp upstream), exons 1–9, and 3′-UTR were identified by genomic PCR with Sanger sequencing (in 13 Greyhounds) or by analysis of publicly available whole genome sequence data by sampling one dog from each of 45 different breeds. Samples from Greyhound dogs were identified by their owners as dogs registered with the National Greyhound Association bred for racing. Shown are the locations of each polymorphism, predicted effect on the cDNA and protein, as well as the observed allele frequencies (95% confidence interval) in the Greyhounds and the dogs from the other breeds. Genotype data for each individual dog used to derive these allele frequencies are given in S1 Table. The genetic polymorphism labels used here (#1 to #9) correspond to the labels used in Fig. 4 and Table 3.

^a^Position in base pairs in the CanFam 3.1 chromosome 1 sequence for the first nucleotide of the polymorphism.

### *CYP2B11* haplotype analysis

Linkage disequilibrium analysis indicated strong linkage across the *CYP2B11* gene (spanning about 16 kilobases) for most polymorphisms in both Greyhounds (Fig. 4a) and dogs from 45 other breeds (Fig. 4b). Exceptions were c.2498G/T, which was associated only with the exon 7 SNP and partially with the 5′-enhancer polymorphism, while the 3′-UTR SNP c.1952 C/T was not associated with any of the other polymorphisms.

Six haplotypes (designated *CYP2B11*-H1 to -H6) could be inferred from genotype data for all dogs (listed in Table 3). Three haplotypes were found in Greyhounds (*CYP2B11*-H1, H2 and H3). *CYP2B11*-H1 and –H2 were the two most common haplotypes found in both Greyhounds and other dog breeds, although *CYP2B11*-H2 predominated (50% frequency) in Greyhounds, while *CYP2B11*-H1 predominated (62% frequency) in other breeds. The other haplotype found in Greyhounds (*CYP2B11*-H3) was much more common in Greyhounds (19% frequency) compared with other breeds (3% frequency). Apart from Greyhounds, *CYP2B11*-H3 was found in a Whippet (homozygous) and a Border Collie (heterozygous *CYP2B11*-H1/H3).

**Table 3:** *CYP2B11* haplotypes in Greyhounds and other dog breeds. Greyhounds (n = 13) and one dog from each of 45 other breeds were genotyped for 9 polymorphisms in the *CYP2B11* gene.

	Genetic polymorphism									Haplotype frequency % (N haplotype/N total haplotypes)	
	#1	#2	#3	#4	#5	#6	#7	#8	#9	Greyhounds (n = 13)	Other breeds (n = 45)
Haplotype 1	G	**A**	TCA	C	TG	G	A	**T**	G	31 (8/26)	62 (56/90)
Haplotype 2	**A**	G	**TCCA**	C	**CA**	**A**	**G**	**T**	**C**	50 (13/26)	18 (16/90)
Haplotype 3	G	**A**	TCA	**T**	TG	G	A	**T**	G	19 (5/26)	3 (3/90)
Haplotype 4^a^	G	G	TCA	C	TG	G	A	G	G	—	8 (7/90)
Haplotype 5	**A**	G	TCA	C	TG	G	A	G	G	—	7 (6/90)
Haplotype 6	G	G	**TCCA**	C	TG	G	A	**T**	G	—	2 (2/90)

Samples from Greyhound dogs were identified by their owners dogs registered with the National Greyhound Association bred for racing. Details regarding the polymorphisms are given in Table 2. Six haplotypes (H1 to H6) could be inferred from these genotypes. The allele sequences are shown for each haplotype. Alleles that differ from the CanFam 3.1 reference sequence are indicated by bolding and underlining for each haplotype. Also shown are the frequencies of each haplotype. The genetic polymorphism labels used here (#1 to #9) correspond to the labels used in Fig. 4 and Table 2.

^a^Haplotype 4 was identical to the CanFam 3.1 reference sequence.

### Breed heterogeneity in *CYP2B11* H2 and H3 haplotype frequencies

The heterogeneity of the *CYP2B11*-H2 and -H3 haplotypes across breeds was evaluated in greater depth by genotyping DNA sampled from 64 different breeds (minimum 10 dogs per breed), including 19 Sighthound breeds, 45 other (non-Sighthound) breeds, and 153 mixed-breed dogs. Greyhound samples (n = 241) included 180 National Greyhound Association (NGA)-registered dogs bred for racing and 61 dogs bred for other purposes registered with the American Kennel Club (AKC).

An initial comparison (Table 4) of haplotype frequencies between breeds that comprised the liver samples studied above (i.e. Beagles, NGA-registered Greyhounds, and mixed-breed dogs) showed similar H2 frequencies across the three breeds (21–26%), but a much higher H3 frequency in NGA-registered Greyhounds (18%) compared with mixed-breed dogs (2%). The H3 haplotype was not found in any genotyped Beagle dog samples. Interestingly, AKC-registered Greyhounds were quite different from the NGA-registered Greyhounds in that they lacked the H2 haplotype and had the highest H3 frequency of all breeds sampled (59%).

**Table 4:** Comparison of *CYP2B11* haplotype frequencies in Greyhound, Beagle and mixed-breed dogs.

Breed	N dogs	Number of dogs with each CYP2B11 diplotype						Haplotype frequency (%)	
		H1/H1	H1/H2	H2/H2	H1/H3	H2/H3	H3/H3	H2	H3
Beagle	50	30	15	5	0	0	0	25	0
Mixed-breed	153	95	44	9	3	2	0	21	2
Greyhound (NGA)	180	56	54	12	39	14	5	26	18
Greyhound (AKC)	61	14	0	0	22	0	25	0	59

DNA samples were genotyped by allelic discrimination assay for haplotype-specific polymorphisms, including c.2137 TG/CA (*CYP2B11*-H2) and c.1952 C/T (*CYP2B11*-H3). Shown are the numbers of dogs with each diplotype and the derived haplotype frequencies. Greyhounds were divided into two groups based on whether they were identified by their owners as dogs registered with the National Greyhound Association (NGA) bred for racing or dogs registered with the American Kennel Club (AKC) bred for other purposes.

**Figure 5:**
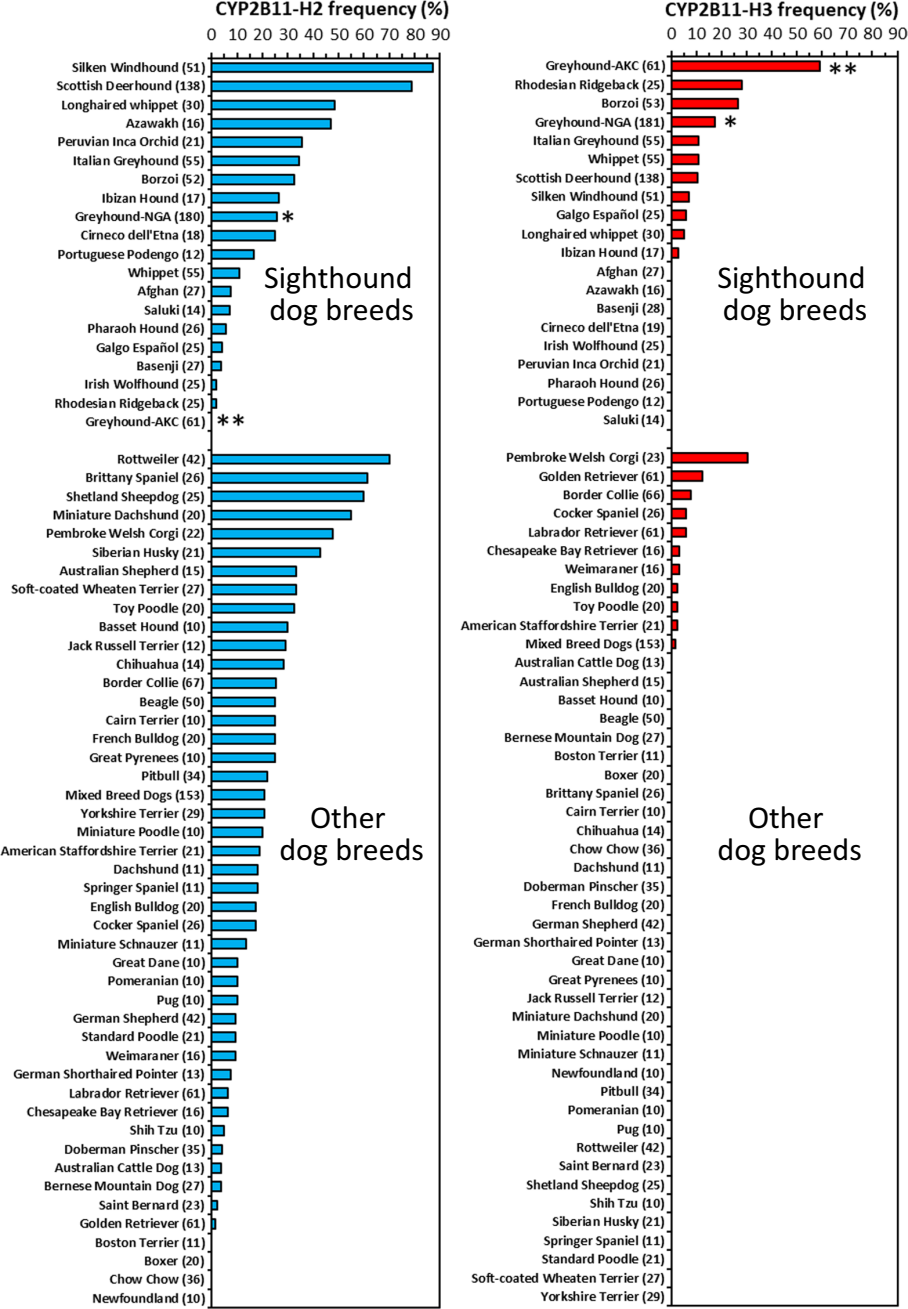
Breed variation in *CYP2B11* haplotypes in Sighthounds and other breeds. *CYP2B11*-H2 and -H3 genotypes were determined using 2,057 DNA samples collected from 64 different breeds, including 19 Sighthound breeds, 45 other (non-Sighthound) breeds, and 153 mixed-breed dogs. Breeds were designated by the dog’s owner. Greyhounds were divided into two breed sub-groups based on whether they were identified by their owners as dogs registered with the National Greyhound Association (NGA*) bred for racing or were dogs registered with the American Kennel Club (AKC**) bred for other purposes. Haplotype frequencies are shown for individual breeds grouped into “Sighthound dog breeds” and “Other dog breeds” for comparison. Shown next to the breed name are the number of individual dogs that were sampled. At least 10 dogs were sampled per breed.

A broader evaluation of haplotype frequencies across Sighthound and non-Sighthound breed groups is shown in Fig. 5. The H2 haplotype was widely distributed across most breeds and was detected in all 19 (100%) of the Sighthound breeds sampled as well as in 41 of 45 (91%) non-Sighthound breeds. Furthermore, average (±SE) H2 frequency calculated for the breed groups was similar (P > 0.05, Mann-Whitney *U* test) in Sighthound (25 ± 6%) compared with non-Sighthound breeds (20 ± 3%). On the other hand, the H3 haplotype was more restricted in breed distribution, being found in 10 of 19 (53%) Sighthound breeds and only 10 of 45 (22%) non-Sighthound breeds. Furthermore, average haplotype frequency in Sighthound breeds (9 ± 3%) was over 4-fold higher (P = 0.003, Mann-Whitney *U* test) compared with non-Sighthound breeds (1.7 ± 0.7%).

### *CYP2B11* mRNA splicing

To explore the mechanism underling *CYP2B11* expression variability, whole transcriptome sequencing (RNA-seq) analysis was conducted using total RNA extracted from the same 5 Greyhound and 5 Beagles livers used for determining CYP activities and *CYP2B11* mRNA quantitation to evaluate variation in mRNA splicing of *CYP2B11* gene transcripts. Mapping with transcript analysis identified only a single transcript in all samples that was identical in mRNA length exon structure to the *CYP2B11* reference sequence in Genbank (NM_001006652). No alternate splice forms were found.

### *CYP2B11* mRNA allelic imbalance

**Figure 6:**
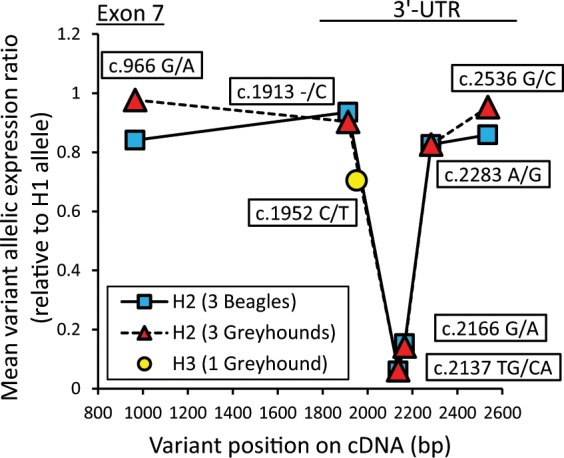
*CYP2B11* mRNA allelic imbalance. Variant allelic expression ratios were derived by RNA-seq analysis of liver RNA from dogs that were identified as heterozygous for the H1/H2 (3 Beagles and 3 Greyhounds) and H1/H3 (one Greyhound) diplotypes. Raw ratios (averaged by breed and diplotype group) were corrected for mapping efficiency differences between alleles by using whole genomic sequencing data obtained from H1/H2 and one H1/H3 diplotype dogs. Details are given in the Materials and Methods section. Corrected allelic expression ratios are shown plotted against the polymorphism position in the cDNA (adenine in start codon = +1). Samples from Greyhound dogs were identified by their owners as dogs registered with the National Greyhound Association bred for racing.

A potential role for *cis*-acting regulatory genetic polymorphisms in *CYP2B11* gene expression was evaluated by assessment of allelic imbalance using RNA-seq data for a subset of the previously studied liver samples that were found to be heterozygous with *CYP2B11*-H1 for the *CYP2B11*-H2 allele (3 Beagles and 3 Greyhounds) and for the *CYP2B11*-H3 allele (1 Greyhound). To account for mapping efficiency differences, RNA allelic ratios at each variant position were normalized using DNA allelic ratios obtained from whole genome DNA sequence data for 5 other dogs with the *CYP2B11*-H1/H2 genotype and one other dog with the *CYP2B11*-H1/H3 genotype. As shown in Fig. 6, dramatically lower RNA expression (mean ratios of 0.05 to 0.15) was observed for the *CYP2B11*-H2 allele relative to the *CYP2B11*-H1 allele for 2 of the 6 polymorphisms (c.2137 TG/CA and c.2166G/A) in both Greyhound and Beagle livers. *CYP2B11*-H3 expression was slightly lower (ratio of 0.7) than *CYP2B11*-H1 at the single SNP (c.1952 C/T) associated with this haplotype.

### *CYP2B11* 3′-UTR haplotype reporter gene expression

**Figure 7:**
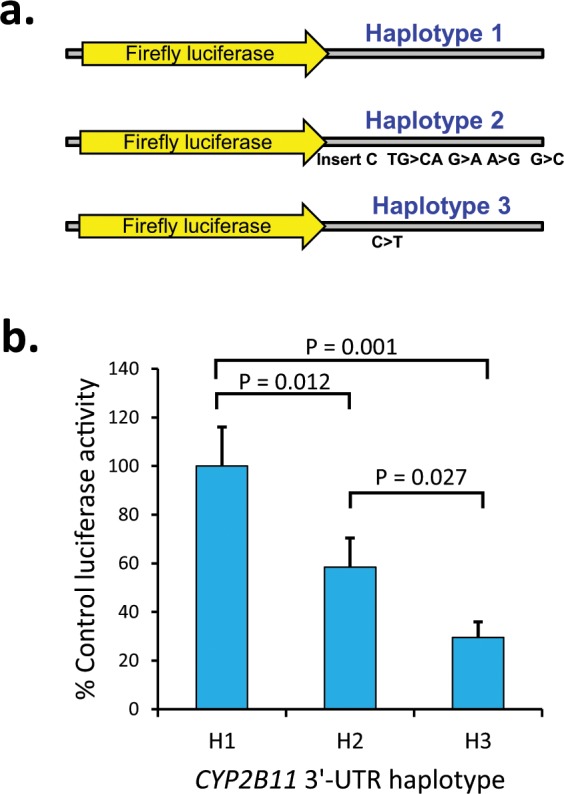
Effect of *CYP2B11* 3′-UTR polymorphisms on gene expression. (**a**) Firefly luciferase 3′-UTR reporter plasmids were constructed using the complete 3′UTR region cloned using DNA from dogs homozygous for the H1, H2 and H3 haplotypes. (**b**) Plasmids were co-transfected with Renilla luciferase (transfection control) into MDCK cells and assayed using the Dual-Glo assay kit. Results are expressed relative to *CYP2B11*-3′UTR-H1 plasmid transfected cells and represent the mean (standard deviation) of 3 independent experiments conducted in quadruplicate. Significant differences between haplotypes were identified by ANOVA (*P* < 0.05). Shown are the P-values for *post hoc* pairwise multiple comparisons testing to H1 control (Holm-Sidak method).

The effect on gene expression of a subset of the *CYP2B11*-H2 and *CYP2B11*-H3 polymorphisms located in the 3′-UTR region were then evaluated using 3′-UTR-luciferase reporter constructs transiently transfected into canine MDCK cells. The constructs (illustrated in Fig. 7a) included *CYP2B11*-3′UTR-H1 (control), *CYP2B11*-3′UTR-H2 (c.1913 TCA > TCCA; c.2137 TG > CA; c.2166G > A; c.2283A > G; c.2536G > C) and *CYP2B11*-3′UTR-H3 (c.1952 C > T). As shown in Fig. 7b, compared to *CYP2B11*-3′UTR-H1, *CYP2B11*-H3-3′-UTR showed markedly lower gene expression (by over 70%; P = 0.001, Holm-Sidak test), while expression of *CYP2B11*-H2-3′-UTR was intermediate (about 40% less than *CYP2B11*-H1-3′UTR; P = 0.012, Holm-Sidak test).

### *CYP2B11* 3′-UTR transcript length variation

**Figure 8:**
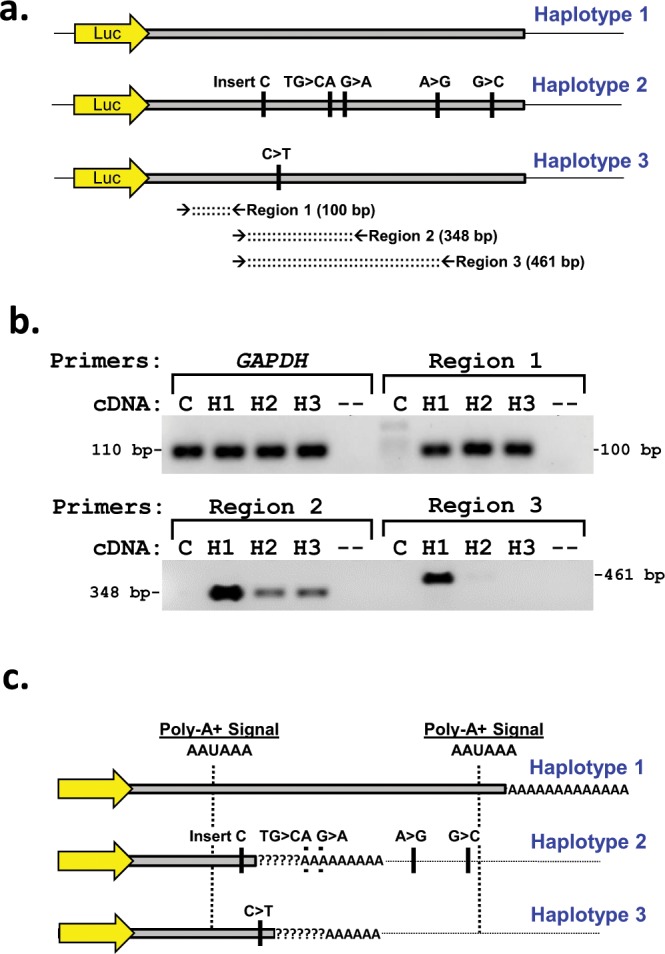
Effect of *CYP2B11* 3′-UTR polymorphisms on transcript length. (**a**) PCR primers were designed to amplify *CYP2B11* 3′-UTR cDNA upstream (5′) of any polymorphism (Region 1) and downstream (3′) adjacent to Region 1 overlapping two polymorphisms (c.2137 TG/CA and c.2166 G/A) that demonstrated significant allelic imbalance (Regions 2 and 3). (**b**) PCR was then conducted with each primer set using reverse transcribed RNA extracted from MDCK cells transfected with luciferase reporter constructs containing each of the *CYP2B11* 3′-UTR haplotypes (H1, H2 or H3), untransfected cells (C), or no input RNA (−). PCR primers for the housekeeping gene, *GAPDH*, were included to exclude an effect of differences in RNA extraction and reverse transcription efficiency. DNA bands of the appropriate size were identified by agarose gel electrophoresis with Sybr Green staining. Supplementary Figure S1 contains full length gels. (**c**) Sequence analysis of the *CYP2B11* 3′-UTR identified two canonical consensus polyadenylation signal (AAUAAA) sites. Their locations are indicated relative to the predicted 3′ ends of each 3′-UTR haplotype. (?) indicates the region likely to contain the 3′-end of the mRNA based on RT-PCR and RNA-seq data.

Reverse transcriptase PCR was used to determine the approximate length of the 3′ end of the *CYP2B11*-3′UTR reporter mRNA in MDCK cells transfected with each of the luciferase reporter constructs. PCR primers were designed to amplify the cDNA in 3 regions, from position c.1773 to c.1872 (Region 1), from c.1853 to c.2199 (Region 2) and from c.1853 to c.2312 (Region 3) (Fig. 8a). These regions were chosen to be upstream (5′) of the two polymorphisms (c.2137 TG/CA and c.2166G/A) that demonstrated significant allelic imbalance (Region 1), or to span these polymorphisms (Regions 2 and 3). Primers for *GAPDH* were also used to confirm RNA extraction and reverse transcription in each sample. Untransfected cells were assayed to exclude background *CYP2B11* expression in the cell line.

As shown in Fig. 8b, the *GAPDH* primers resulted in bands of similar intensity in cells transfected with each *CYP2B11*-3′UTR reporter construct, as well as in untransfected cells. Strong bands were also detected with the Region 1 primers for all three *CYP2B11*-3′UTR reporter constructs, but not in untransfected cells. The Region 2 primers resulted in a strong band for *CYP2B11*-3′UTR-H1, but much weaker bands for *CYP2B11*-3′UTR-H2 and *CYP2B11*-3′UTR-H3. Furthermore, the Region 3 primers showed a strong band for *CYP2B11*-3′UTR-H1, but no bands for *CYP2B11*-3′UTR-H2 or *CYP2B11*-3′UTR-H3.

By combining the RT-PCR results with the RNA-seq allelic imbalance information, the approximate locations of the 3′ end of the mRNA for each *CYP2B11* polymorphism were inferred (shown in Fig. 8c). For *CYP2B11*-3′UTR-H1, the data were consistent with the 3′end at c.2625 as given in the Genbank reference sequence NM_001006652. For *CYP2B11*-3′UTR-H2, the 3′end is likely located between c.1913 and c.2138, while for *CYP2B11*-3′UTR-H3, it is likely between c.1952 and c.2199.

The *CYP2B11* 3′-UTR sequence was then evaluated for the presence of consensus polyadenylation signal sites. Two canonical polyadenylation signal sites (AAUAAA) were found. One site was located at c.2582, about 40 bp upstream of the predicted 3′ end of *CYP2B11*-3′UTR-H1, while the other site was at c.1715, about 200 bp upstream of the predicted ends of *CYP2B11*-3′UTR-H2 and *CYP2B11*-3′UTR-H3. None of the 3′-UTR polymorphisms appeared to create a novel consensus polyadenylation signal site or abolish an existing one.

### *CYP2B11* diplotype association with activity, protein and mRNA

**Figure 9:**
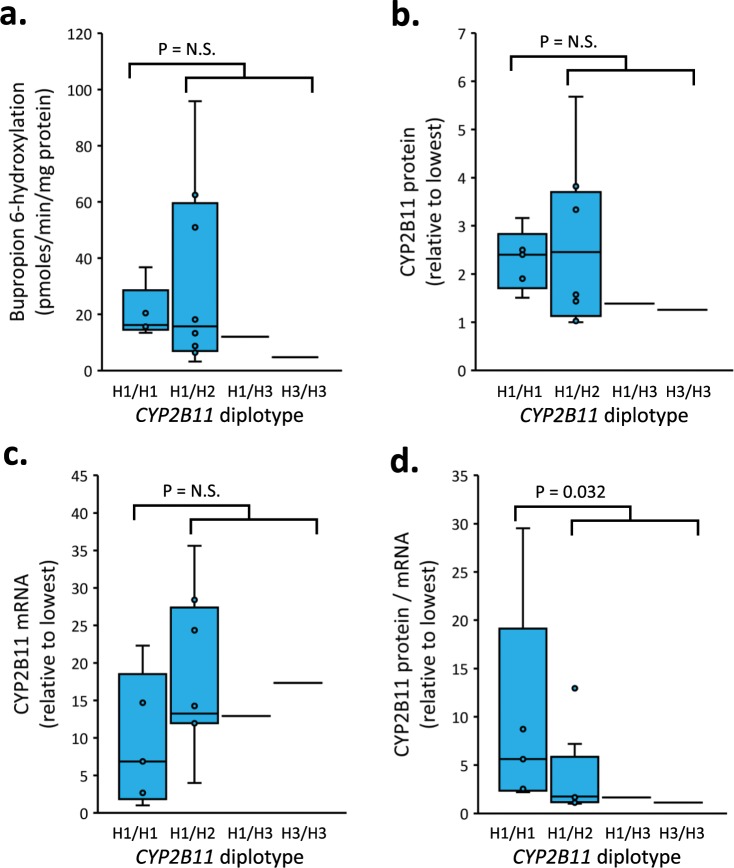
*CYP2B11* diplotype-phenotype association analysis. Differences in (**a**) CYP2B11 activity, (**b**) protein, (**c**) mRNA, and (**d**) protein/mRNA ratio between the 15 previously studied dog livers are shown as box and whisker plots after grouping by *CYP2B11* diplotype. Diplotypes included H1/H1 (2 Beagles and 3 mixed-breed), H1/H2 (3 Beagles, 2 mixed-breed, and 3 Greyhounds), H1/H3 (one Greyhound) and H3/H3 (one Greyhound). No dogs had the H2/H2 diplotype. Since only one dog liver had the H1/H3 diplotype and one liver had the H3/H3 diplotypes, these data were grouped with the H1/H2 livers (10 livers total) for statistical comparison by Mann-Whitney *U* test (P-values shown, N.S = not statistically significant) with the H1/H1 livers (5 livers). Samples from Greyhound dogs were identified by their owners as dogs registered with the National Greyhound Association bred for racing.

Differences in CYP2B11 enzyme activity, protein content, mRNA abundance, and protein/mRNA ratio (as an index of translation efficiency) between the 15 (previously studied) dog livers after grouping by *CYP2B11* diplotype are shown in Fig. 9. Identified diplotypes included H1/H1 (2 Beagles and 3 mixed-breed), H1/H2 (3 Beagles, 2 mixed-breed, and 3 Greyhounds), H1/H3 (one Greyhound) and H3/H3 (one Greyhound). No dogs possessed the H2/H2 diplotype. Since there was only one dog liver with the H1/H3 diplotype and one dog liver with the H3/H3 diplotype, these data were grouped with the H1/H2 livers (10 livers total) for statistical comparison with the H1/H1 livers (5 livers). No differences in bupropion hydroxylation, CYP2B11 protein abundance or *CYP2B11* mRNA expression were observed between H1/H1 and other diplotypes (P > 0.05, Mann-Whitney *U* test). However, CYP2B11 protein/mRNA values were significantly higher (P = 0.032, Mann-Whitney *U* test) in the H1/H1 group, compared with livers with other diplotypes with median (interquartile range) ratios of 5.6 (2.4–19) and 1.7 (1.1–3.2).

## Discussion

Based on the results of the microsomal CYP activity marker assays and CYP2B11 immunoblotting, this study provides further evidence that CYP2B11 is deficient in Greyhounds. Furthermore, other CYPs involved in drug metabolism appear to be equally active, or, in the case of CYP2D15, perhaps even more active in Greyhounds compared with other dog breeds. Recombinant enzyme phenotyping indicated that propofol hydroxylation is largely mediated by CYP2B11, although with some involvement from CYP3A12. A role for CYP3A12 in propofol hydroxylation was confirmed by showing significant correlation of propofol hydroxylation activities with microsomal CYP3A protein content, although somewhat weaker than with CYP2B11 protein content. To rule out possible, perhaps additional, deficiency of CYP3A12 in Greyhounds, breed differences were evaluated using activity probes that were confirmed by recombinant enzyme phenotyping to be more selective than propofol hydroxylation for CYP2B11 (bupropion hydroxylation) and CYP3A12 (omeprazole sulfonation). Results using these latter probes suggest that CYP3A12 is not deficient in Greyhounds. These results have since been confirmed by us through quantitation of microsomal CYP protein concentrations using proteomic techniques that are more accurate and precise than immunoblotting^15^.

*CYP2B11* mRNA concentrations in Greyhound livers were similar to Beagle livers and higher than mixed-breed livers indicating that low CYP2B11 activity and protein content in Greyhound livers was not a consequence of reduced gene transcription or mRNA instability, but could involve aberrant mRNA splicing or reduced translational efficiency. The most clinically important genetic polymorphism in human *CYP2B6* (g.15631G/T) is located within a splice enhancer site in exon 4 that results in exon skipping and exclusion of exons 4, 5 and 6 from the final edited transcript^16^. However, evaluation of the *CYP2B11* liver transcriptome by RNA-seq analysis excluded mRNA splicing variation as a potential mechanism in Greyhounds.

mRNA allelic imbalance analysis has been used for a number of years in pharmacogenetic research and related disciplines to identify *cis*-acting polymorphisms that differentially alter expression levels of mRNA transcribed from different alleles^17^. Samples that are known to be heterozygous at polymorphic sites located within the transcript are typically used to enable direct comparison of the amount of variant transcript with the reference transcript within the same sample. Here, we used RNA-seq data from liver samples that were heterozygous for polymorphisms located within the *CYP2B11* transcript. These polymorphisms had been identified by sequencing of genomic DNA extracted from the same liver samples and included 6 linked variants located in exon 7 and the 3′-UTR (*CYP2B11*-H2), and one SNP in the 3′UTR (*CYP2B11*-H3).

Although there was no clear evidence for allelic imbalance with the *CYP2B11*-H3 SNP, we did observe almost complete loss of expression of the variant allele at two, but not all 6 of the *CYP2B11*-H2 variant sites. This finding was identical in all 6 liver samples with the H1/H2 diplotype (regardless of breed). Since these two variants (c.2137 TG/CA and c.2166G/A) were located within the middle 3′-UTR region, this finding is consistent with a shortened 3′-UTR in the *CYP2B11*-H2 mRNA at some position between c.1913 and c.2137, rather than at the expected 3′-UTR end at c.2625. This was confirmed by RT-PCR analysis of the transfected *CYP2B11*-H1 and *CYP2B11*-H2 3′-UTR luciferase reporters (Fig. 7b). Furthermore, in the initial study that cloned *CYP2B11* cDNA, two *CYP2B11* mRNA species (one long and one short) by Northern blot analysis of dog liver RNA were also reported^18^. The approximate sizes of those mRNA species (2.9 kb and 1.9 kb) are similar to the predicted sizes of the *CYP2B11*-H1 (2.6 kb) and *CYP2B11*-H2 (1.9 – 2.1 kb) mRNA. Finally, sequence analysis of the 3′-UTR identified two canonical consensus polyadenylation signal sites, one located upstream of the *CYP2B11*-H1 3′-end and an alternate canonical polyadenylation signal site upstream of the predicted *CYP2B11*-H2 3′-end (Fig. 7C).

Surprisingly, we did not observe *CYP2B11*-H2 allelic imbalance in the RNA-seq data at the two polymorphic sites further downstream (c.2283A/G and c.2636G/C). This would have been expected if the shorter *CYP2B11*-H2 transcript was generated through early termination of transcription with polyadenylation close to the internal alternate polyadenylation signal site. Recently, a novel widely used mechanism has been identified that generates shorter transcripts from longer transcripts through post-transcriptional 3′-UTR cleavage^19^. This process results in two separate RNA fragments; the mRNA coding region with a shorter 3′-UTR tail and a stable uncapped autonomous RNA fragment. Our RNA-seq data provides preliminary evidence that such a mechanism may be involved in generating the shorter (final) *CYP2B11*-H2 transcript from a longer (precursor) *CYP2B11*-H2, as well as a separate stable RNA fragment containing the variant allele. Importantly, our data suggests that one or more of the *CYP2B11*-H2 3′-UTR polymorphisms may serve to enhance utilization of this process through mechanisms that do not involve altering the polyadenylation signal sequence.

Since the *CYP2B11*-H3 only consisted of a single 3′-UTR SNP located at c.1952, RNA-seq data was uninformative regarding the length of the *CYP2B11*-H3 3′UTR downstream of this position. However, RT-PCR of *CYP2B11* 3′-UTR luciferase reporters indicated that the *CYP2B11*-H3 3′UTR was also truncated relative to *CYP2B11*-H1 with a length that was similar to *CYP2B11*-H2. More precise mapping of the 3′UTR of the *CYP2B11* mRNA variants could be done in futures studies using techniques such as 3′-rapid amplification of cDNA ends (3′-RACE) or single molecule real-time (SMRT) sequencing.

The main purpose of constructing the *CYP2B11* 3′-UTR luciferase reporters was to evaluate the functional effects of the H2 and H3 haplotypes on gene expression. Both haplotypes significantly reduced gene expression as measured by luciferase activity, although H3 had the greatest effect, more than twice that of H2. Truncation of the 3′-UTR in the H2 and H3 variants would be expected to decrease mRNA stability. However, no differences were observed in mRNA expression between H1, H2 and H3 luciferase constructs using primers targeting Region 1 (Fig. 8B). Furthermore, *CYP2B11* mRNA abundance was not lower in dog livers with either of the H2 or H3 haplotypes compared to those with only the H1 haplotype (Fig. 9C). Genotyped dog liver data did suggest that these haplotypes might reduce translational efficiency as reflected by lower CYP2B11 protein/mRNA ratios (Fig. 9D). Consequently, it is possible that the *CYP2B11*-H2 and *CYP2B11*-H3 variants create novel binding sites for microRNAs on the mature mRNA (c.1913 insert C and c.1952 C > T, respectively), which are known to regulate gene expression by repressing translation.

Although Greyhounds are the principle breed reported to experience anesthetic drug sensitivity, veterinarians, owners, and breeders suspect that some closely related breeds within the Sighthound group of dog breeds may also be sensitive^4–6^. Consequently, we determined and compared the prevalence of both the H2 and H3 haplotypes across diverse breeds. We hypothesized that any variant contributing to the slow metabolizer phenotype should be more prevalent in Greyhounds and possibly other related Sighthounds compared with non-Sighthound breeds. This hypothesis was not confirmed for *CYP2B11*-H2, which could reflect the milder effects of this haplotype on drug metabolism phenotype. However, we did find a significantly higher prevalence of *CYP2B11*-H3 among Sighthounds compared with non-Sighthounds, with AKC Greyhounds having the highest H3 frequency of all breeds sampled (nearly 60%).

A recent genomic study indicates that most Sighthound breeds belong to one of two monophyletic groups^20^. Sighthound Group 1 contains the following breeds; Greyhound, Whippet, Scottish Deerhound, Irish Wolfhound, Borzoi, and Italian Greyhound. Sighthound Group 2 contains breeds from most of the other Sighthounds sampled in our study, as well as some breeds not considered Sighthounds, such as Great Pyrenees, Komondor, and Anatolian Shepherd. The CYP2B11-H3 haplotype was found in all of the Sighthound Group 1 breeds except Irish Wolfhound, while only one of the 7 Sighthound Group 2 breeds sampled (Ibizan Hound) had this haplotype. This finding suggests that *CYP2B11*-H3 may have arisen in a common ancestor of the Sighthound Group 1 breeds. Given the sporadic presence of *CYP2B11*-H3 in largely unrelated breeds outside of Group 1, it is likely that *CYP2B11*-H3 was dispersed from the Sighthound Group 1 breeds to other breeds through admixture and haplotype sharing, as was recently shown for other alleles by Parker *et al*.^20^.

AKC Greyhounds differed considerably from NGA Greyhounds in that they had a higher *CYP2B11*-H3 prevalence and lacked *CYP2B11*-H2. This difference may reflect a founder effect that occurred when these two populations were initially isolated. It might also be a consequence of selective breeding for different purposes, in that NGA Greyhounds are primarily bred for racing speed, while AKC Greyhounds are primarily bred for conformation.

Our results predict that some, but not all Greyhounds would have decreased CYP2B11 expression. Lowest CYP2B11 expression would be expected in dogs with the *CYP2B11* H3/H3 diplotype, about 70% lower than for dogs with the H1/H1 diplotype. Although prior reports have shown lower clearance of propofol and thiobarbiturates in Greyhounds compared with mixed-breed dogs, all results were presented as aggregated data (i.e. mean ± SD) from 10 to 12 dogs per breed group^7,8^. Consequently, it is unclear whether there were differences between individual Greyhounds of a magnitude that would be consistent with the difference predicted by our *in vitro* data. It should also be pointed out that non-genetic factors such as enzyme induction and inhibition could contribute to variable CYP2B11 metabolism on top of genetic regulation. This is exemplified by enhancement of thiopental clearance by phenobarbital and inhibition of propofol clearance by chloramphenicol, respectively, in Greyhounds^9,10^.

In addition to detecting *CYP2B11*-H3 in 9 Sighthound breeds (other than Greyhounds), we also found this haplotype in 10 non-Sighthound breeds suggesting that the Sighthound CYP2B11 poor metabolizer phenotype might be found in non-Sighthound breeds. For most of these non-Sighthound breeds, the H3 haplotype frequency was relatively low (less than 10%). Therefore, the predicted frequency of the poor metabolizer CYP2B11 H3/H3 diplotype would be less than 1% (assuming we had a sufficiently representative sample of these breeds). However, we note that three of the breeds, including Labrador Retriever, Golden Retriever, and English Bulldog were ranked by the AKC in 2018 as the first, third, and fifth most popular dog breeds owned in the USA, respectively, based on annual AKC registration^21^. Consequently, the overall impact of this gene variant on these breeds could be substantial, at least in terms of the absolute numbers of dogs affected.

There were some limitations to the current study. The numbers of available dog livers from different breeds and with different genotypes were somewhat limited and so the results utilizing those samples should be viewed with caution. Also, the numbers of available DNA samples for some dog breeds was limited by availability, with a minimal sample size of 10 dogs arbitrarily set by us, so extrapolation to the entire breed should be done with caution. Furthermore, genotyping of the over 2,000 dogs was carried out using single haplotype marker polymorphisms and so for H2, which consists of multiple SNPs, it remains possible that the selected marker is not unique to the variant haplotype within the larger population. Finally, our predictions concerning the impact of the H2 and H3 variants on CYP2B11 expression are entirely based on *in vitro* studies with extrapolation *in vivo*. Consequently, future studies are needed to confirm these findings such as through evaluation of CYP2B11 function *in vivo* using isoform specific drug phenotyping probes comparing dogs from different breeds and with different *CYP2B11* genotypes. Studies are ongoing in our laboratory to further characterize the impact of *CYP2B11* haplotypes *in vivo*.

## Materials and Methods

### Animal ethics statement

The collection and use of liver tissue employed in this study were considered exempt from review by the Institutional Animal Care and Use Committee at Washington State University since all tissues collected would have been normally discarded. The collection, storage, and use of the DNA samples employed in this study were approved by the Institutional Animal Care and Use Committee at Washington State University (protocols #04194 and #04539) and were collected in accordance with relevant guidelines and regulations. Informed owner consent was obtained for all dogs prior to DNA collection.

### Chemicals and reagents

Phenacetin, acetaminophen, 2-acetamidophenol, coumarin, 7-hydroxy-comuarin (umbelliferone), flurbiprofen, dextromethorphan, dextrorphan, trazodone, thymol, bupropion, 6-hydroxy-bupropion, alprazolam, 1′-hydroxy-alprazolam, NADP^+^, isocitrate dehydrogenase, and DL-isocitrate were purchased from Sigma-Aldrich (St. Louis, MO, USA). Pronethalol was from Tocris (Minneapolis, MN, USA). Propofol was provided by Zeneca Pharmaceuticals (Wilmington, DE, USA). 4′-hydroxy-flurbiprofen, 2-fluoro-4-biphenyl-acetic acid, 4-hydroxy-propofol, chlorzoxazone, and 6-hydroxy-chlorzoxazone were purchased from Toronto Research Chemicals (Toronto, ON, Canada). *S*-Mephenytoin and 4-hydroxymephenytoin were purchased from Gentest (Corning, Corning, NY, USA). GW340416A, a chemical analogue of bupropion, was kindly provided by GlaxoSmithKline (Research Triangle Park, NC, USA). Omeprazole was purchased from BeanTown Chemical, Inc. (Hudson, NH, USA) and omeprazole sulfone was purchased from Cayman Chemical Company (Ann Arbor, MI, USA). Sodium hydroxide, potassium phosphate monobasic and potassium phosphate dibasic and EDTA were purchased from J.T. Baker (Center Valley, PA, USA). HPLC-grade acetonitrile and methanol were purchased from Thermo Fisher Scientific (Waltham, MA, USA). Ultra-pure water was obtained using a Milli-Q^®^ Q-POD Millipore System (EMD Millipore, Burlington, MA, USA).

### Dog liver tissues and microsomes

Snap frozen liver tissue samples were obtained and stored at −80 °C from 15 untreated healthy adult dogs, including 5 Greyhounds (3 males and 2 females; all registered NGA dogs bred for racing), 5 male mixed-breed dogs, and 5 male Beagle dogs. Dogs were untreated (control) research animals that had been euthanized for reasons unrelated to this study. Liver microsomes were prepared from the liver tissue samples detailed above as previously described^22^ and stored at −80 °C until use. Microsomal protein concentrations for liver microsomes were determined using the bicinchoninic acid assay (Thermo Fisher Scientific).

### Dog breed DNA sampling

Stored DNA samples from client-owned dogs were retrieved from the Washington State University Veterinary Teaching Hospital Patient DNA Bank (n = 1,182) and the Comparative Pharmacogenomics Laboratory Sighthound DNA Bank (n = 875). DNA had been extracted from buccal swab samples obtained by the hospital staff or by the dog’s owner. The majority of the hospital patient samples derived from dogs living in the Pacific Northwest of the United States, while the Sighthound samples were obtained primarily by mail from dogs living throughout the United States. A dog’s breed was identified by the owner for the Hospital Bank whereas breed was identified by the owner along with accompanying breed registration identification for the Sighthound Bank. For the purposes of this study, the designations “mix”, “mixed”, “cross”, “mutt”, “mongrel” or similar by the owner was considered as a single group of “mixed-breed” dogs. The 2,057 DNA samples represented 64 different dog breeds including 19 Sighthound breeds, 45 non-Sighthound breeds, as well as 153 mixed-breed dogs. The designation of a breed as belonging to the ‘Sighthound’ group was based on the AKC’s breed inclusion for Sighthounds^23^. Samples from Greyhound dogs were divided into two groups based on whether they were identified by their owners as dogs bred for racing and registered with the NGA (n = 180) or were dogs bred for other purposes and registered with the AKC (n = 61). All breed groups included samples from at least 10 different dogs.

### Recombinant canine CYPs

Recombinant canine CYP1A1, CYP1A2, CYP2B11, CYP2C21, CYP2C41, CYP2D15, CYP3A12 and CYP3A26, all co-expressed with canine P450 oxidoreductase (POR) as bactosomes, were purchased from Sekisui Xenotech LLC (Kansas City, KS, USA). Since recombinant canine CYP2A13, CYP2A25 and CYP2E1 were not commercially available, these enzymes were made in-house as follows.

cDNA sequences for canine CYP2A13, CYP2A25, CYP2E1 and POR (NCBI entries NM_001037345.1, NM_001048027.1, NM_001003339.1, and NM_001177805.1, respectively) were synthesized and cloned into the pFastBac1™ vector (Thermo Fisher Scientific) by GenScript (Piscataway, NJ, USA). CYP and POR recombinant baculoviruses were created using the Bac-to-Bac^®^ baculovirus expression system (Thermo Fisher Scientific) following the manufacturer’s protocols. Briefly, recombinant baculoviruses were created by transforming DH10Bac competent *Escherichia coli* with the recombinant pFastBac1™ plasmids using heat shock. Recombinant bacmid DNA was isolated using a QIAprep^®^ Spin Miniprep Kit (Qiagen, Hilden, Germany) and transfected into Sf9 (*Spodoptera frugiperda*) insect cells through Cellfectin^®^ II reagent-mediated gene transfer to produce recombinant baculoviruses. Recombinant baculoviruses were clarified and amplified to create high-titer passage stocks. Gel electrophoresis and DNA sequencing confirmed the presence of the cDNA in recombinant baculovirus. Amplified viral stocks were titered relative to the recombinant POR baculovirus stock using a TaqMan^®^ gene expression assay (Thermo Fisher Scientific) as described by Hitchman *et al*.^24^.

Sf9 shaking suspension cultures were grown in the dark at 27 °C in Sf-900™ II serum-free medium (Thermo Fisher Scientific) supplemented with 5% fetal bovine serum (HyClone Laboratories, Logan, UT, USA) to a cell density of 1.5 × 10^6^ cells/mL. Cells were then co-infected with recombinant viruses encoding CYP and POR at optimal CYP:POR viral ratios determined in preliminary experiments. At 24 h post-infection, hemin (prepared by dissolving in 50% ethanol and 0.2 M NaOH) was added to the culture to achieve a final concentration of 2 µg/mL^25^. Cells were harvested at 72 h post-infection by centrifugation and washed twice with 4 °C phosphate buffered saline (pH 7.4). Cells were stored at −80 °C until use.

Microsomes were prepared by homogenization using a pestle tissue grinder follow by 2-speed centrifugation (9,000 and 100,000 × g at 4 °C) and then reconstituted in 100 mM phosphate buffer (pH 7.4), 20% glycerol and 1 mM EDTA^26^. Functional CYP content of recombinant microsomes was measured by CO-difference spectrum using a microplate assay as described by Yang *et al*.^27^. For the CO-difference spectra, an extinction coefficient (Δ_ε450-490_) of 106,000 M^−1^ cm^−1^ ^28,29^ was used. POR activity of the recombinant microsomes was assessed by the cytochrome c reduction assay as described by Guengerich *et al*.^29^ but scaled to fit a microplate format. Functionality of the recombinant microsomes was assessed through 7-ethoxycoumarin metabolism to umbelliferone (7-hydroxycoumarin) as detailed by Waxman and Chang^30^. Microsomes were stored at −80 °C until use.

### Liver and recombinant CYP enzyme activities

Enzyme activities selective for human CYPs were measured and compared using Greyhound, Beagle and mixed-breed dog liver microsomes (n = 5 livers per breed). Activities (and the corresponding human CYPs) included phenacetin *o*-deethylation (CYP1A), coumarin 7-hydroxylation (CYP2A6), bupropion 6-hydroxylation (CYP2B6), flubiprofen 4-hydroxylation (CYP2C9), (*S*)-mephenytoin 4-hydroxylation (CYP2C19), dextromethorphan *o*-demethylation (CYP2D6), chlorzoxazone 6-hydroxylation (CYP2E1), and omeprazole sulfonation (CYP3A)^31^. There is also evidence that some of these activities are selective for the respective canine CYP ortholog; including phenacetin *O*-deethylation (canine CYP1A)^14^, coumarin 7-hydroxylation (canine CYP2A13)^32^, propofol 4-hydroxylation (canine CYP2B11)^13^, dextromethorphan *O*-demethylation (canine CYP2D15)^33^, and chlorzoxazone 6-hydroxylation (canine CYP2E1)^34^.

*In vitro* incubation assay conditions including substrate concentration, microsomal protein concentrations, incubation time, and analytical method details are given in Supplementary Table S2. Most metabolite concentrations were determined by HPLC with absorbance or fluorescence detection (700-series Satellite Wisp auto-injector, 500-series pump, 486 absorbance detector, 470 fluorescence detector; Waters, Milford, MA, USA). 6-Hydroxybupropion and omeprazole sulfone concentrations were determined using a liquid chromatography-triple-quadrupole (LC-MS/MS) system (Agilent 1100 liquid chromatography system; Agilent Technologies, Inc., Santa Clara, CA, USA connected to an API 4000 mass spectrometer, AB Sciex, Framingham, MA, USA). Preliminary studies were conducted for each biotransformation using pooled dog liver microsomes to ensure linear metabolite formation with respect to increasing time and microsomal protein concentration. The rate of metabolite formation was calculated by dividing the metabolite concentration in the sample by the incubation time and microsomal protein concentration. Experiments were conducted in duplicate and results for individual liver microsomes were averaged. Propofol 4-hydroxylation activity values reported previously for the same set of dog liver microsomes^13^ were also used to compare to these newly generated data.

Propofol 4-hydroxylation, bupropion 6-hydroxylation, and omeprazole sulfonation activities were also measured using a panel of 11 recombinant canine CYP enzymes that included CYPs 1A1, 1A2, 2A13, 2A25, 2B11, 2C21, 2C41, 2D15, 2E1, 3A12, and 3A26. Propofol 4-hydroxylation activities were quantified as previously described^12,13^ with slight modifications as follows. Recombinant CYP concentration in the incubation was 10 pmol/mL, propofol concentration was 5 μM, while the incubation time was 10 min. The HPLC column used was a 4 μm, 150 × 2 mm Phenomenex^®^ Synergi™ Fusion-RP 80 Å (Torrance, CA, USA). A gradient mobile phase (total flow of 0.4 mL/min) was used consisting of mobile phases A (100% acetonitrile) and B (80% 20 mM phosphate buffer and 20% acetonitrile, v/v). The gradient was as follows: linear gradient from 10 to 20% A over 10 min, 20 to 46% A over 10 min, 46 to 100% A over 2 min, 100 to 10% A over 1 min. Bupropion 6-hydroxylation and omeprazole sulfonation activities were quantified as described in Supplementary Table S2 for liver microsomes, except that recombinant CYP enzymes were used instead of liver microsomes at an incubation concentration of 10 pmol/mL. The rate of metabolite formation was calculated by dividing the final metabolite concentration by the incubation time and recombinant CYP concentration. Unless otherwise indicated, all experiments were performed in duplicate and results were averaged for the data point. All experiments were repeated at least three times on separate days.

The relative contributions of individual CYP isoforms to total liver microsome propofol hydroxylation, bupropion hydroxylation, and omeprazole sulfonation activities were estimated by adjustment of specific CYP activities using the average liver microsome abundance of each CYP. Abundance values determined by mass spectrometry in liver microsomes from 59 dogs of differing breeds were 2.8, 82, 11, 7.7, 79, 52, 1.8, 143, 72, 125, and 3.8 pmoles CYP per mg microsomal protein for CYPs 1A1, 1A2, 2A13, 2A25, 2B11, 2C21, 2C41, 2D15, 2E1, 3A12, and 3A26, respectively^15^.

### CYP1A, CYP2B11, and CYP3A protein content by immunoblotting

Microsomal CYP1A, CYP2B11 and CYP3A protein content were determined by semi-quantitative immunoblotting using the same Greyhound, Beagle and mixed-breed dog liver microsomes (n = 5 per breed) described above. The technique was based on a method described previously with minor modifications^35^. Rabbit polyclonal antisera raised against rat CYP1A2 (AB1255) and rat CYP3A1 (AB1253) were purchased from Chemicon Millipore (Temecula, CA, USA). Rabbit polyclonal antisera raised against dog CYP2B11 was a generousgift from Dr. James Halpert (School of Pharmacy, University of Connecticut, Storrs, CT, USA)^36^. Briefly, 10 µg of microsomal protein was separated by sodium dodecyl sulfate acrylamide gel electrophoresis using a 26-well 5 to 15% gradient gel (Criterion, Biorad, Hercules, CA, USA). Proteins were then electrophoretically transferred using a semi-dry technique to polyvinyl difluoride membrane (Immobilon-P; Millipore Corporation). Membranes were blocked in 5% powdered nonfat milk in Tris-buffered saline-Tween (0.15 M NaCl, 0.04 M Tris, pH 7.7, and 0.1% Tween 20) for one hour at room temperature and then incubated overnight at 4 °C in Tris-buffered saline-Tween/5% milk containing the primary antibody at an appropriate dilution (1:500 for CYP1A2; 1:6,000 for CYP2B11; 1:1,000 for CYP3A). Blots were washed, reblocked, and then incubated at room temperature for one hour with a 1:10,000 dilution of a goat anti-rabbit IgG antibody conjugated to horse radish peroxidase (PerkinElmer, Inc., Waltham, MA, USA). After washing, chemiluminescence reagent (Super Signal; Pierce Chemical Cp., Dallas, TX, USA) was applied, and blots were imaged using the Kodak Image Station 440CF (Kodak, Rochester, NY, USA). Bands were quantified using Kodak 1D Image Analysis Software (Kodak) and net intensity values for each liver sample were expressed relative to the liver sample containing the lowest band intensity. Final results for each liver sample represent the average of 3 independent experiments. Preliminary studies were conducted using serial dilutions of pooled dog liver microsomes to ensure a linear relationship between the amount of microsomal protein loaded and band intensity up to 20 µg of loaded protein. To ensure equal protein loading, membranes were washed and total protein was visualized with Ponceau S reagent^37^.

### Liver *CYP2B11* mRNA quantitation

Total RNA was isolated using TRIZOL Reagent (ThermoFisher Scientific) from the same Greyhound, Beagle and mixed-breed dog livers (n = 5 per breed) used to isolate microsomes. *CYP2B11* mRNA content relative to *18S* rRNA content was determined by real-time PCR with Sybr Green-based detection (CFX96 Touch, Bio-Rad) as previously described^38^. Primers for *CYP2B11* mRNA were Pri_459_forward: 5′-GGA TTC AGG AGG AGG CTC AGT GTC-3′ and Pri_460_reverse 5′-GAT GTT GGC GGT CAT GGA GTG G. Primers for *18S* rRNA were Pri_127_forward: 5′-CCC CTC GCT GCT CTT AGC TGA GTG T-3′ and Pri_128_reverse 5′-CGC CGG TCC AAG AAT TTC ACC TCT.

### *CYP2B11* sequencing and genotyping

Genetic polymorphisms located in the *CYP2B11* 5′-enhancer (to ~2,000 bp upstream), exons 1–9, and 3′-UTR were identified by Sanger sequencing of genomic PCR product using DNA obtained from 13 Greyhounds (5 from liver samples and 8 from buccal swab samples). Primers used for PCR and sequencing, as well as the gene region amplified and product size are given in Supplementary Table S3 Table. Genotype data from the same *CYP2B11* gene regions were also obtained from another 45 dogs (each of a different breed) by analysis of publicly available whole genome sequence data. Briefly, binary alignment files originally submitted by the Institute of Genetics, University of Bern, Switzerland were downloaded from the European Nucleotide Archive (Study ID PRJEB16012). Polymorphisms were identified and genotypes called on individual dog samples using the Freebayes bayesian genetic variant detector (arXiv:1207.3907) as implemented in Galaxy version 1.1.0^39^ on a Bioteam Appliance (Bioteam, Middleton, MA, USA). The IDs of individual dogs that were sequenced and analyzed, as well as their nominal breed, are listed in Supplementary Table S1. Haploview^40^ was used to evaluate the extent of linkage disequilibrium between identified polymorphic sites across the *CYP2B11* gene and to resolve individual haplotypes.

Custom allele discrimination assays (Applied Biosystems TaqMan SNP Genotyping Assay, Thermo Fisher Scientific) were used to genotype DNA samples from 2,057 dogs for the *CYP2B11* haplotype marker polymorphisms c.2137 TG/CA (CYP2B11-H2) and c.1952 C/T (CYP2B11-H3). Primer and reporter sequences are given in Supplementary Table S4. Assays were performed using a real-time PCR instrument (CFX96 Touch, Bio-Rad).

### *CYP2B11* RNA-seq

RNA-seq was conducted as described previously^41^. Total RNA was extracted from the same Greyhound and Beagle livers (n = 5 per breed) used for determining CYP activities and quantifying *CYP2B11* mRNA. Briefly, cDNA libraries were prepared from total RNA from each liver using the Truseq Stranded Total RNA LT kit (Illumina, San Diego, CA, USA). Libraries were sequenced on an Illumina 2000 Instrument at the Columbia Genome Center (New York, NY, USA), generating 60 million 100-bp paired-end reads. After quality filtering, reads were mapped to the canine reference genome (CanFam3.1) using Tophat version 1.5.0^42^ and transcripts assembled using Cufflinks version 0.0.7^42^, as implemented in Galaxy version 1.1.0^39^ on a Bioteam Appliance (Bioteam). For allele expression analysis, mapped read depths of the variant and reference alleles in heterozygous H1/H2 and H1/H3 samples at the site of each polymorphism comprising the H2 and H3 haplotypes were obtained using GenomeBrowse version 2.1.2 (Golden Helix, Bozeman, MT, USA). Variant to reference ratios were obtained for each dog and averaged by breed and diplotype group. These raw average ratios were then corrected for possible mapping efficiency differences between alleles by dividing by ratios obtained at the same polymorphic sites using mapped DNA sequences from whole genomic sequencing data (described above). These included 5 (other) H1/H2 diplotype dogs and a H1/H3 dog (since only one dog could be identified with this diplotype).

### *CYP2B11*-3′-UTR luciferase reporter assay

Plasmid luciferase 3′UTR reporter constructs containing the entire *CYP2B11*-3′UTR reference (-H1) and variant (-H2 and -H3) haplotypes were created using methods previously described with minor modifications^43^. Briefly, PCR was performed using DNA from dogs that were homozygous for the *CYP2B11*-3′UTR H1, H2, and H3 haplotypes. Primers were Pri_1183_forward: 5′-GAC AAC TAG TGA GGG TGC TGA GGG AAG G-3′ and Pri_1184_reverse 5′-GAC AAA GCT TAT GGC TCA CCA CCT GAC C-3′, which contain 5′-end *Hind III* and *Spe I* sites, respectively. PCR products were purified and cloned into the *Hind III* and *Spe I* sites in the pMIR-REPORT vector. Plasmid clone insert sequences were verified by Sanger sequencing. The transfection host was a canine kidney cell line (MDCK [NBL-2] [ATCC^®^ CCL-34™], ATCC, Manassas, VA, USA) grown in Eagles minimum essential medium (Thermo-Fisher Scientific) supplemented with 10% fetal bovine serum (HyClone Laboratories). Approximately 50,000 cells/well were seeded onto 96-well clear bottom, white-sided, tissue culture treated plates (Corning) one day prior to transfection. Cells were co-transfected with 6 ng of the *CYP2B11*-3′UTR plasmids and 2 ng of the renilla luciferase pRL-CMV transfection plasmid (Promega, Madison, WI, USA) with Lipofectamine 2000 reagent (Thermo Fisher Scientific). Cells were assayed for luciferase and renilla activities 48 h after transfection using the Dual-Glo assay kit (Promega) following the manufacturer’s protocol on a SpectraMax i3 plate reader operated with Softmax Pro 6.3 software (Molecular Devices, San Jose, CA, USA). Each transfection was carried out in four wells and results were averaged. Independent experiments were repeated on three different days. The final results for each variant *CYP2B11*-3′UTR haplotype were expressed as the mean (and standard deviation) percent of control (H1) renilla normalized luciferase activities.

Reverse transcription followed by PCR (RT-PCR) was used to evaluate effects of the *CYP2B11*-3′UTR-H2 and *CYP2B11*-3′UTR-H3 variants compared with *CYP2B11*-3′UTR-H1 (control) on expressed RNA in this cell model. Briefly, total RNA was extracted (Qiagen spin column) from cells harvested 48 h after transfection. RNA (200 ng) was treated with DNAse I enzyme (Thermo Fisher Scientific) and reverse transcribed (Multiscribe, Thermo Fisher Scientific) using random hexamer primers (Thermo Fisher Scientific). cDNA was then amplified by PCR for 35 cycles using to the manufacturers recommended method (Platinum Taq Supermix, Thermo Fisher Scientific) on a thermal cycler (C1000, Biorad). PCR primer sets are given in Supplementary Table S5. PCR products (10 µL) were run on a 1.8% agarose gel stained with Sybr Green and images recorded (Biorad Chemidoc MP).

### Statistical analyses

Statistical analyses were performed using SigmaPlot 13 software (Systat Software Inc., San Jose, CA, USA). Associations between breed and drug metabolizing activities and CYP2B11 protein and mRNA content were evaluated by analysis of variance (ANOVA) on log-transformed data with *post-hoc* pairwise testing by Holm-Sidak multiple comparisons test. Student’s *t*-test on log transformed data was also used to evaluated differences in enzyme activities between Greyhound and non-Greyhound liver microsomes. Relationships between CYP immunoreactive protein content and CYP activities were determined by calculation of the Spearman’s correlation coefficient. *CYP2B11* haplotype effects on luciferase activity were determined by ANOVA followed by Holm-Sidak multiple comparisons test. *CYP2B11* diplotype effects on CYP2B11 activity, protein, mRNA and protein abundance normalized to mRNA expression in the liver bank, as well as haplotype frequencies between Sighthound and non-Sighthound breed groups were evaluated by Mann-Whitney *U* test. For all statistical tests, a P-value < 0.05 was considered statistically significant.

## Supplementary information

Supplementary Information.

## Data Availability

All data generated and analyzed during this study are included in this published article and its Supplementary Information files.
