# Supplementary material for: Pharmacogenomics of poor drug metabolism in Greyhounds: Cytochrome P450 (CYP) 2B11 genetic variation, breed distribution, and functional characterization
Source: Sci Rep. 2020 Jan 9;10:69. doi: 10.1038/s41598-019-56660-z (PMC6952448; doi:10.1038/s41598-019-56660-z)
Supplement: Supplementary file 1 — Supplementary Information. [file 41598_2019_56660_MOESM1_ESM.pdf]

Pharmacogenomics of poor drug metabolism in Greyhounds: Cytochrome P450 (CYP) 2B11 genetic variation, breed distribution, and functional characterization

Stephanie E. Martinez[*], Marie C. Andresen, Zhaohui Zhu, Ioannis Papageorgiou[#], and Michael H. Court

Comparative Pharmacogenomics Laboratory, Program in Individualized Medicine (PrIMe), Department of Veterinary Clinical Sciences, College of Veterinary Medicine, Washington State University, Pullman, Washington, United States of America
*s_martinez@wsu.edu
[#]Current Address: Department of Pathology, Immunology and Laboratory Medicine, College of Medicine, Diabetes Institute, University of Florida, Gainesville, Florida, United States of America

Supplementary Information

Figure S1. Full-length gels of cropped gels displayed in Figure 8B.

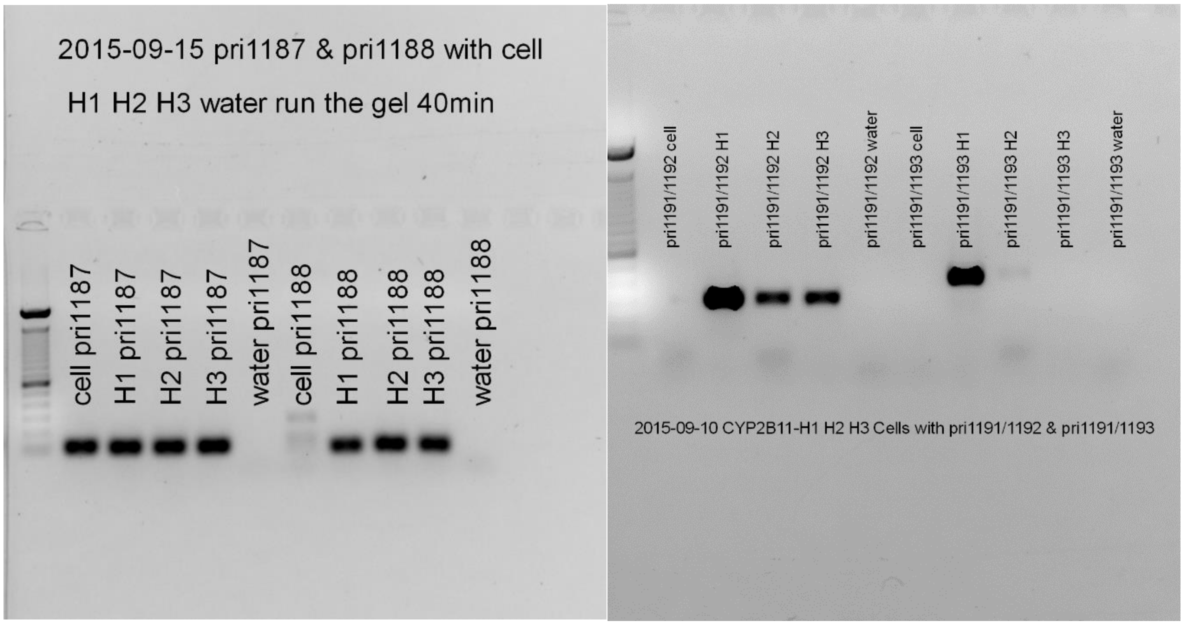

Table S1.

		CYP2B11 polymorphism genotypes								
Dog ID	Breed	c.-489 G/A	c.966 G/A	c.1913 TCA/TCCA	c.1952 C/T	c.2137 TG/CA	c.2166 G/A	c.2283 A/G	c.2498 G/T	c.2536 G/C
DL08	Greyhound	G/A	G/A	TCA/TCCA	C/C	TG/CA	G/A	A/G	T/T	G/C
DL10	Greyhound	G/G	A/A	TCA/TCA	C/T	TG/TG	G/G	A/A	T/T	G/G
DL09	Greyhound	G/A	G/A	TCA/TCCA	C/C	TG/CA	G/A	A/G	T/T	G/C
DL03	Greyhound	G/G	A/A	TCA/TCA	T/T	TG/TG	G/G	A/A	T/T	G/G
DL04	Greyhound	G/A	G/A	TCA/TCCA	C/C	TG/CA	G/A	A/G	T/T	G/C
MC14-430	Greyhound	G/A	G/A	TCA/TCCA	C/C	TG/CA	G/A	A/G	T/T	G/C
MC14-431	Greyhound	G/A	G/A	TCA/TCCA	C/C	TG/CA	G/A	A/G	T/T	G/C
MC14-432	Greyhound	G/A	G/A	TCA/TCCA	C/C	TG/CA	G/A	A/G	T/T	G/C
MC14-437	Greyhound	G/G	A/A	TCA/TCA	T/T	TG/TG	G/G	A/A	T/T	G/G
MC14-438	Greyhound	G/A	G/A	TCA/TCCA	C/C	TG/CA	G/A	A/G	T/T	G/C
MC14-441	Greyhound	A/A	G/G	TCCA/TCCA	C/C	CA/CA	A/A	G/G	T/T	C/C
MC14-445	Greyhound	A/A	G/G	TCCA/TCCA	C/C	CA/CA	A/A	G/G	T/T	C/C
MC14-485	Greyhound	A/A	G/G	TCCA/TCCA	C/C	CA/CA	A/A	G/G	T/T	C/C
AC108	Australian Cattle dog	G/A	G/A	TCA/TCCA	C/C	TG/CA	G/A	A/G	T/T	G/C
AM007	Alaskan Malamute	G/G	G/A	TCA/TCA	C/C	TG/TG	G/G	A/A	G/T	G/G
AR001	Australian Terrier	G/G	A/A	TCA/TCA	C/C	TG/TG	G/G	A/A	T/T	G/G
AS006	American Staffordshire Terrier	G/G	G/A	TCA/TCA	C/C	TG/TG	G/G	A/A	G/T	G/G
BB011	Berger Blanc Suisse	G/G	A/A	TCA/TCA	C/C	TG/TG	G/G	A/A	T/T	G/G
BC0480	Border Collie	G/A	G/A	TCA/TCCA	C/C	TG/CA	G/A	A/G	T/T	G/C
BD052	Bearded Collie	G/G	A/A	TCA/TCA	C/C	TG/TG	G/G	A/A	T/T	G/G
BG064	Bavarian Hound (Bayerischer Gebirgsschweisshund)	G/G	A/A	TCA/TCA	C/C	TG/TG	G/G	A/A	T/T	G/G
BH003	Basset	G/G	A/A	TCA/TCA	C/C	TG/TG	G/G	A/A	T/T	G/G
BT007	Miniature Bullterrier	A/A	G/G	TCCA/TCCA	C/C	CA/CA	A/A	G/G	T/T	C/C
BU002	Bull Mastiff	G/A	G/A	TCA/TCCA	C/C	TG/CA	G/A	A/G	T/T	G/C
CE073	Cairn Terrier	G/G	A/A	TCA/TCA	C/C	TG/TG	G/G	A/A	T/T	G/G
CK006	Cavalier King Charles Spaniel	G/A	G/A	TCA/TCCA	C/C	TG/CA	G/A	A/G	T/T	G/C
CP003	Cocker Spaniel	G/G	A/A	TCA/TCA	C/C	TG/TG	G/G	A/A	T/T	G/G
CR039	Curly Coated Retriever	G/G	A/A	TCA/TCA	C/C	TG/TG	G/G	A/A	T/T	G/G
CW011	Chow Chow	G/G	A/A	TCA/TCA	C/C	TG/TG	G/G	A/A	T/T	G/G
DD116	Great Dane	G/G	G/A	TCA/TCCA	C/C	TG/TG	G/G	A/A	T/T	G/G
DH0117	Dachshund	G/A	G/G	TCA/TCCA	C/C	TG/CA	G/A	A/G	G/T	G/C
DS043	German Shepherd	G/A	G/A	TCA/TCCA	C/C	TG/CA	G/A	A/G	T/T	G/C

EL565	Elo	G/G	A/A	TCA/TCA	C/C	TG/TG	G/G	A/A	T/T	G/G
EN091	Entlebucher Sennenhund	G/A	G/A	TCA/TCCA	C/C	TG/CA	G/A	A/G	T/T	G/C
EU035	Eurasier	G/A	G/A	TCA/TCA	C/C	TG/TG	G/G	A/A	G/T	G/G
FB065	French Bulldog	G/G	A/A	TCA/TCA	C/C	TG/TG	G/G	A/A	T/T	G/G
GR0855	Golden Retriever	G/A	G/A	TCA/TCA	C/C	TG/TG	G/G	A/A	G/T	G/G
GR1078	Golden Retriever Mix	G/G	A/A	TCA/TCA	C/C	TG/TG	G/G	A/A	T/T	G/G
HT001	Heideterrier	G/G	G/A	TCA/TCA	C/C	TG/TG	G/G	A/A	G/T	G/G
HW1706	Hovawart	G/A	G/A	TCA/TCCA	C/C	TG/CA	G/A	A/G	T/T	G/C
LA1869	Labrador Retriever	G/G	A/A	TCA/TCA	C/C	TG/TG	G/G	A/A	T/T	G/G
LB1919	Leonberger	A/A	G/G	TCCA/TCCA	C/C	CA/CA	A/A	G/G	T/T	C/C
LN47	Border Collie	G/G	A/A	TCA/TCA	C/T	TG/TG	G/G	A/A	T/T	G/G
LR753	Lagotto Romagnolo	G/G	A/A	TCA/TCA	C/C	TG/TG	G/G	A/A	T/T	G/G
MA008	Malinois	G/G	G/A	TCA/TCA	C/C	TG/TG	G/G	A/A	G/T	G/G
NW062	Norwich Terrier	G/A	G/A	TCA/TCA	C/C	TG/TG	G/G	A/A	G/T	G/G
PL116	Poodle	G/G	G/A	TCA/TCA	C/C	TG/TG	G/G	A/A	G/T	G/G
RR123	Rhodesian Ridgeback	A/A	G/G	TCA/TCA	C/C	TG/TG	G/G	A/A	G/G	G/G
SG006	Sloughi	A/A	G/G	TCCA/TCCA	C/C	CA/CA	A/A	G/G	T/T	C/C
SH008	Greater Swiss Mountain Dog	G/G	G/A	TCA/TCA	C/C	TG/TG	G/G	A/A	G/T	G/G
SL006	Saluki	G/G	G/A	TCA/TCCA	C/C	TG/TG	G/G	A/A	T/T	G/G
SS004	Shetland Sheepdog	A/A	G/G	TCCA/TCCA	C/C	CA/CA	A/A	G/G	T/T	C/C
SY046	Siberian Husky	G/G	A/A	TCA/TCA	C/C	TG/TG	G/G	A/A	T/T	G/G
TA001	Airedale Terrier	G/G	A/A	TCA/TCA	C/C	TG/TG	G/G	A/A	T/T	G/G
WE006	Weimaraner	G/A	G/A	TCA/TCA	C/C	TG/TG	G/G	A/A	G/T	G/G
WH083	Whippet	G/G	A/A	TCA/TCA	T/T	TG/TG	G/G	A/A	T/T	G/G
WW174	West Highland White Terrier	G/G	A/A	TCA/TCA	C/C	TG/TG	G/G	A/A	T/T	G/G
ZS14_2	Pomeranian	G/G	A/A	TCA/TCA	C/C	TG/TG	G/G	A/A	T/T	G/G

Table S1. Genotypes of *CYP2B11* polymorphisms identified in 13 Greyhounds and 45 other dog breeds. Genetic polymorphisms located in the *CYP2B11* 5'-enhancer (to ~2,000 bp upstream), exons 1-9, and 3'-UTR were identified by genomic PCR and Sanger sequencing (13 Greyhounds) or by analysis of publicly available whole genome sequence data (one dog from each of 45 different breeds). Samples from Greyhound dogs were identified by their owners as dogs registered with the National Greyhound Association (NGA) bred for racing.

S2 Table.

Assay	Substrate concentration (µM)	Protein concentration (mg / mL)	Incubation time at 37°C (min)	Internal standard	HPLC column	Mobile phase	Detector
Phenacetin-*o*-deethylation	50	0.5	20	2-acetamido-phenol	µBondapak C18 3.9 x 300 mm	25% acetonitrile in 50 mM aqueous potassium phosphate buffer pH 4.5	Absorbance at 254 nm
Coumarin 7-hydroxylation	1	1	60	Phenacetin	Novapak C18 3.9 x 150 mm	25% acetonitrile and 0.2% phosphoric acid in water	Fluorescence (EX/EM) at 338/458 nm
Bupropion 6-hydroxylation	5	0.5	30	GW340416A	Phenomenex Synergi Fusion-RP 80Å 4 µm, 2 x 150 mm	0.1% formic acid in water with 20-95% acetonitrile as a linear gradient	LC-MS/MS set at 256.3/238 *m/*z (hydroxybupropion) and 238/182 *m/*z (internal standard)
Propofol 4-hydroxylation	5	0.05	10	Thymol	Phenomenex Synergi Fusion-RP 80Å 4 µm, 2 x 150 mm	20 mM potassium phosphate buffer (pH 7.4) in water with 20-95% acetonitrile as a linear gradient	Fluorescence (EX/EM) at 276/310 nm
Flurbiprofen 4-hydroxylation	100	0.5	120	2-fluoro-4-biphenyl-acetic acid	µBondapak C18 3.9 x 300 mm	58% acetonitrile in 20 mM aqueous potassium phosphate buffer pH 3.0	Fluorescence (EX/EM) at 260/320 nm
S-mephenytoin 4-hydroxylation	250	0.5	120	Phenacetin	µBondapak C18 3.9 x 300 mm	20% acetonitrile in 50 mM aqueous potassium phosphate buffer pH 4.5	Absorbance at 204 nm
Dextromethorphan *o*-demethylation	25	0.5	10	Pronethalol	µBondapack C18 3.9 x 300 mm	30% acetonitrile in 50 mM aqueous potassium phosphate buffer pH 4.5	Fluorescence (EX/EM) at 280/310 nm
Chlorzoxazone 6-hydroxylation	100	1	20	Phenacetin	µBondapack C18 3.9 x 300 mm	25% acetonitrile in 50 mM aqueous potassium phosphate buffer pH 4.5	Absorbance at 295 nm
Omeprazole sulfonation	5	0.2	15	Pantoprazole	Agilent ZORBAX Eclipse XDB-C18 5 µm, 2.1 x 50 mm	0.1% formic acid in H20:MeOH:ACN; 90:9:1 to 80:10:10 to 55:22.5:22.5, v/v/v as linear gradient	LC-MS/MS set at 362/150.1 *m/*z (omeprazole sulfone) and 384.2/200.1 *m/*z (internal standard)

S2 Table. CYP activity assays. Details of incubation conditions and analytical assay methods used to measure CYP enzyme activities.

Table S3.

Primer ID	Sequence (5’-3’)	Gene region amplified	Amplicon size (bp)
Pri_1130_forward	TGCAGAGCCAGACATGGAGC	Enhancer (5’)	418
Pri_1131_reverse*	TGCCTTCATGGTAGGACACTTCTC	Enhancer (5’)	418
Pri_1128_forward*	GACACCAAGACAGAGGGATATAGCA	Enhancer (middle)	489
Pri_1129_reverse	GAATGCGCTGATGTGTGAATCTAC	Enhancer (middle)	489
Pri_1126_forward*	CCACCATCACCCAAATGCTAAA	Enhancer (3’)	544
Pri_1127_reverse	ATCAGAAGCAAGAGCCCCGT	Enhancer (3’)	544
Pri_1150_forward*	TAAAAGGCTCAGCTCGAGGCT	Exon 1	272
Pri_1151_reverse	ATACGGAGGCAGGGAGCAAC	Exon 1	272
Pri_1134_forward*	ACTGGTATGGCTGCATAGGTGC	Exon 2	298
Pri_1135_reverse	TCTCTTCCCAAATTCATCCTCTCC	Exon 2	298
Pri_1136_forward*	GGGACACAGGGTCTCCTTCCA	Exon 3	319
Pri_1137_reverse	CCAGGTGCCCTTGGGATCA	Exon 3	319
Pri_1152_forward*	GCAGACACACAGACATGGGGCA	Exon 4	291
Pri_1153_reverse	AGATGTCCACCTCATGCCCACC	Exon 4	291
Pri_1140_forward*	CTCCACTGGGACCCACAGCT	Exon 5	352
Pri_1141_reverse	TTGGCATCCCGTGTCCTTC	Exon 5	352
Pri_1142_forward	TGCGAGAGACCCTGAGGAGG	Exon 6	287
Pri_1143_reverse	AGGACCTCAGCCCCACTTCC	Exon 6	287
Pri_1144_forward*	CATGATGAATGAGCACTGGGTG	Exon 7	469
Pri_1145_reverse	TTCCCTTCCTCAACCCATTTG	Exon 7	469
Pri_1146_forward*	CCAGTCTGGGTGCTCTTAATCTCTG	Exon 8	356
Pri_1147_reverse	GCAAAGATCTCTCCTGGATTGTCTC	Exon 8	356
Pri_1156_forward*	GGACACAGATATATGGGTTTGGGA	Exon 9	372
Pri_1157_reverse	TTCAGTGGCAGGAAGACCCA	Exon 9	372
Pri_915_forward*	TGACCTCACACCCCAGGAGATAGGT	3’-UTR (5’)	654
Pri_916_reverse*	TTGAGTTTGAGAACCAGGGAGACAGAG	3’-UTR (5’)	654
Pri_1124_forward	TCCATGATCTGTGCCTATGTGAGC	3’-UTR (middle)	366
Pri_1125_reverse*	GGGTAAGGGGTTTTCTTCTTGAGTG	3’-UTR (middle)	366
Pri_917_forward*	GGCACTGGCATCAGTTTTCCCA	3’-UTR (3’)	708
Pri_918_reverse*	CCCAGAAAGATCCCTAGTGACCTGACA	3’-UTR (3’)	708

Table S3. *CYP2B11* gene PCR primers. PCR primers used to amplify and sequence the *CYP2B11* gene 5’-enhancer, exons 1-9 and 3’-UTR from canine genomic DNA. *Indicates primers also used to sequence the PCR product.

Table S4.

Assay name	Polymorphism	Sequence (5'-3')	Purpose
CYP2B11-H2	c.2137 TG/CA	CCTAACCACAACCCCCATTATATTTTTAAATTATAGT	Forward primer
		GTGGTGGTGGTTGTACAACATTG	Reverse primer
		VIC-ACAAGTGTTTTAAAG**TG**TATAAT-NFQ	Reporter 1
		FAM-AGTGTTTTAAAG**CA**TATAAT-NFQ	Reporter 2
CYP2B11-H3	c.1952 C/T	GCCTATGTGAGCTCACTTGAATCTT	Forward primer
		CAGTGCCTGGCCTTTGTTT	Reverse primer
		VIC-CTCGTCT**G**TAAAACAC-NFQ	Reporter 1
		FAM-ACTCGTCT**A**TAAAACAC-NFQ	Reporter 2

Table S4. *CYP2B11* haplotype marker primer and reporter sequences used for Taqman allelic discrimination assay.

Table S5.

Primer ID	Sequence (5’-3’)	Target	Amplicon size (bp)
Pri_1187_forward	AACAGTGACACCCACTCTTC	*GAPDH*	110
Pri_1187_reverse	CGGTTGCTGTAGCCAAATTC	*GAPDH*	110
Pri_1188_forward	CAGGTGCTCTCTGCATTTCT	*CYP2B11* 3’-UTR Region 1	100
Pri_1188_reverse	GCACAAATGGTTGCTCAGTG	*CYP2B11* 3’-UTR Region 1	100
Pri_1191_forward	CACTGAGCAACCATTTGTGC	*CYP2B11* 3’-UTR Region 2/3	348/461
Pri_1192_reverse	GAACTCGATAGTGGTGGTGGT	*CYP2B11* 3’-UTR Region 2	348
Pri_1193_reverse	AAGCCACAGAGACTGGAAGC	*CYP2B11* 3’-UTR Region 3	461

Table S5. *CYP2B11*-3’UTR and *GAPDH* (control gene) RT-PCR primers.
